# Mindfulness training and systemic low-grade inflammation in stressed community adults: Evidence from two randomized controlled trials

**DOI:** 10.1371/journal.pone.0219120

**Published:** 2019-07-11

**Authors:** Daniella K. Villalba, Emily K. Lindsay, Anna L. Marsland, Carol M. Greco, Shinzen Young, Kirk Warren Brown, Joshua M. Smyth, Catherine P. Walsh, Katarina Gray, Brian Chin, J. David Creswell

**Affiliations:** 1 Psychology Department, Carnegie Mellon University, Pittsburgh, Pennsylvania, United States of America; 2 Psychology Department, University of Pittsburgh, Pittsburgh, Pennsylvania, United States of America; 3 College of Medicine, University of Vermont, Burlington, Vermont, United States of America; 4 Psychology Department, Virginia Commonwealth University, Richmond, Virginia, United States of America; 5 Department of Biobehavioral Health, Pennsylvania State University, University Park, Pennsylvania, United States of America; Università degli Studi di Milano, ITALY

## Abstract

Mindfulness interventions have garnered significant attention as a complementary health treatment for many physical and psychological conditions. While some research has shown that mindfulness training can decrease psychological and physiological stress responses, it remains unclear whether mindfulness training impacts inflammation—a predictor of poor health outcomes. In addition, little research has examined the active components of mindfulness that may drive health-related improvements. Here, we provide data from two 3-arm randomized controlled trials that examined the effect of mindfulness training on inflammation in stressed community adults. Specifically, we examined whether training individuals to have an accepting attitude towards present moment experiences is a key emotion regulation skill that can lead to decreases in inflammation. Both studies randomly assigned participants to one of three conditions: mindfulness training that taught both attention monitoring and acceptance skills (Monitor+Accept); mindfulness training teaching monitoring without the acceptance component (Monitor Only); or a control condition. Study 1 employed a novel 2-week smartphone-based intervention and Study 2 employed a standard 8-week Mindfulness-Based Stress Reduction (MBSR) intervention. We hypothesized that Monitor+Accept training would lead to reductions in the inflammatory biomarker C-Reactive Protein (CRP) compared to Monitor Only training and control groups. Contrary to this hypothesis, we found that Monitor+Accept mindfulness training did not lead to reductions in CRP. Exploratory analyses combining study subsamples, however, suggest that both mindfulness interventions may reduce CRP in populations at risk for systemic inflammation—midlife-to-older adults and individuals with high BMI. Overall, the present studies contribute significantly to the question of whether mindfulness interventions can reduce systemic markers of low-grade inflammation.

## Introduction

Chronic low-grade inflammation is a predictor of disease-specific morbidity and mortality [1]. Epidemiological studies have consistently shown that elevated levels of the inflammatory biomarker C-Reactive Protein (CRP) are associated with an increase in risk for developing cardiovascular disease, autoimmune and metabolic diseases, Alzheimer’s disease, and cancer [2–5]. Notably, chronic stress has been associated with elevated levels of CRP [6], raising the possibility that stress management interventions such as mindfulness meditation may be effective in reducing inflammation and inflammation-related health risks [7]. Mindfulness meditation–a practice of *monitoring* present experiences with an attitude of *acceptance* [8,9]–has been shown to reduce physiological and subjective experiences of stress [10–12]. However, the available evidence from randomized controlled trials (RCTs) is mixed regarding the effects of mindfulness training on CRP [13]. The present work describes two RCTs that evaluate whether mindfulness interventions reduce CRP among stressed community adults, and examines potential psychological mechanisms for these effects. Specifically, we test whether acceptance skills training is a critical emotion regulation mechanism for reducing stress and thus stress-related health biomarkers [14]. Using dried blood spot (DBS) samples collected at pre- and post-intervention in two pre-registered RCTs, we tested: (1) whether mindfulness interventions reduce circulating levels of CRP in stressed community adults, and (2) whether removing acceptance skills training from mindfulness interventions eliminates the potential benefit for lowering inflammation. Finally, given the heterogeneity in treatment effects found in previous RCTs, we conducted exploratory post hoc analyses to test whether subgroups of individuals known to have higher levels of systemic inflammation derive benefit from mindfulness training.

### Evidence linking mindfulness interventions and inflammation

Despite research findings showing the health-related benefits of mindfulness training [15], the evidence linking mindfulness interventions with markers of systemic low-grade inflammation is mixed. Although one waitlist control RCT found decreases in CRP among non-flared ulcerative colitis patients following an 8-week Mindfulness-Based Stress Reduction (MBSR) course [16], other RCTs–with and without active treatment control groups–have shown non-significant reductions in CRP [17–19]. Marginal reductions in CRP following mindfulness interventions have, however, been observed among overweight adults at risk for cardiovascular disease [20] and among lonely older adults at risk for all-cause mortality [21]. Collectively, this work suggests that mindfulness interventions may reduce inflammation among at-risk populations who have elevated baseline levels of inflammation or who are at high-risk for inflammatory diseases, but that mindfulness training may not be sufficient to lower inflammation among stressed but otherwise healthy adults.

### The role of acceptance in mindfulness training

Until recently, little empirical attention has focused on how the components of mindfulness may affect health. Monitor and Acceptance Theory (MAT) posits that the two primary components of mindfulness interventions, *attention monitoring*–an ongoing awareness of present moment experiences (e.g., thoughts, feelings, body sensations) and *acceptance*–the ability to attend to such experiences with non-judgment, openness, and receptivity, are dissociable regulatory skills that play specific roles on improving psychological and physical health. Specifically, MAT argues that developing acceptance in conjunction with attention monitoring skills fosters non-reactivity to stressful experiences and buffers individuals against the negative outcomes of stress [14]. As evidence, one study showed that a higher trait tendency to monitor present-moment experience was associated with lower blood pressure and lower circulating IL-6 only among those who also reported high trait acceptance [22]. Still, very few studies have experimentally tested the role of acceptance training in mindfulness interventions. Promisingly, data from a 2-week smartphone-based dismantling RCT (same sample reported in Study 1) showed that mindfulness training in both attention monitoring and acceptance skills buffered against physiological stress reactivity compared to both a structually matched mindfulness program that did not include acceptance training and an active treatment control [10]. This work provides initial support that acceptance skills training may be an important mechanism for mindfulness intervention effects on stress-related health biomarkers, such as CRP.

A more nuanced understanding of the mechanistic components of mindfulness interventions will continue to help improve the quality and effectiveness of mindfulness interventions. Using an experimental mindfulness intervention dismantling approach, we present evidence from two RCTs that delivered different doses of mindfulness training to stressed community adults and examined the role of acceptance training on circulating levels of CRP. Study 1 used a novel 2-week mindfulness training smartphone application to examine the effect of a brief, remote intervention on CRP. Study 2 used a standardized 8-week, in-person MBSR course to examine the effects of a larger-dose mindfulness intervention program on CRP. Together, these two well-controlled RCTs tested different mindfulness intervention doses and delivery methods while dismantling theoretically-guided mechanisms of mindfulness training (monitoring, acceptance). We predicted that learning both monitoring and acceptance skills would lead to significant decreases in circulating CRP compared to learning monitoring skills only or compared to a control intervention or a no treatment control. Given evidence suggesting that mindfulness interventions reduce markers of inflammation among patient populations and those at high risk for inflammatory disease [16,20,21], we explored the primary hypotheses among higher-risk participants only (overweight and obese stressed adults; midlife-to-older stressed adults) in a combined Study 1 and 2 dataset.

## Overview of studies

### Inclusion and exclusion criteria

Both trials used participant registries, community advertisements, and mass emails to local organizations to recruit stressed, community-dwelling adults from the Pittsburgh area. Eligibility requirements were as follows: fluent English speaker; between the ages of 18 and 70; moderate to high levels of stress as indicated by a score above five on the 4-item Perceived Stress Scale [23–25]; ownership of a data-enabled smartphone; and no current systematic mindfulness meditation or related mind-body practice (>2 times per week). We excluded individuals with medical conditions and medication use that may interfere with hypothalamic-pituitary-adrenal (HPA) axis or immune system functioning; who had been hospitalized in the past three months; had a chronic mental or physical disease; current antibiotic, antiviral, or antimicrobial treatment; current oral contraceptive use or who were pregnant; reported using recreational drugs; reported excessive alcohol use; and/or had traveled to countries on the Center for Disease Control (CDC) travel alert list in the past six months. In addition, participants were re-scheduled if they reported more than mild cold or flu symptoms (e.g., sore throat, sneezing, congestion, cough, runny nose, chills, headache, or feeling under the weather) over the past 24 hours given that the common cold and flu are associated with elevated inflammation [26].

## General procedure

Studies 1 and 2 were run concurrently, the 2-week smartphone-based intervention included an active treatment and the 8-week MBSR intervention included a no treatment control. Study procedures were approved by the Carnegie Mellon Institutional Review Board. Interested participants underwent a phone screening to determine initial eligibility. Those who met the inclusion criteria were invited to our research lab for an in-person screening and baseline assessment between 2:00pm and 6:00pm. After confirming eligibility, enrolled participants completed a battery of tasks and questionnaires, and provided a dried blood spot (DBS) sample. This procedure involves pricking the finger pad with a sterile lancet and dropping blood onto a protein collection paper. The samples are allowed to air dry and are subsequently frozen at -20 C until batch analysis [27]. Then, participants received instructions about the training program and study activities. Participants were randomized by an unblinded study statistician into one of three study conditions using a random number generator in a 3-3-2 randomization sequence: A Monitor+Accept training program, a Monitor Only training program, or a control group. Participants completed three consecutive days of ambulatory assessments on their smartphone before and after the intervention. After the intervention, participants returned to our research lab for a post-treatment assessment session at approximately the same time as their baseline appointment (within 1 hour) where they completed the same questionnaires and task battery, provided a second DBS sample, completed the Trier Social Stressor Task (TSST), received compensation, and were fully debriefed. This paper reports results from the assay of CRP from DBS samples. Results from the TSST and ambulatory assessments are reported elsewhere [10,28–30].

## Study 1: Remote 2-week smartphone application mindfulness intervention

Study 1 was pre-registered with Clinical Trials identifier NCT02433431. This manuscript describes changes in circulating CRP from pre- to post-2-week intervention period. All outcome measures reported here were not analyzed until data collection was complete.

## Method

### Participants

A total of 153 stressed community adults (67% female; *M*_*age*_ = 32, *SD* = 14) enrolled in the study. The majority of participants were White (53%), followed by Black (22%), Asian (22%), and Bi- or Multi-Racial (4%). Data collection occurred between February 2015 and April 2016. For this 2-week smartphone intervention, sample size was calculated for primary stress reduction outcomes in the larger trial [10]. These effect size estimates were based on medium-large effects of a pre-post 8-week mindfulness intervention compared to waitlist control on stress reactivity outcomes (*d* = .63; [12]) and small-medium effects of a pre-post 2-week online mindfulness intervention compared to a waitlist control on general stress perceptions (*d* = .37-.46; [31,32]). Therefore, we estimated a medium effect size (*d* = .52) for this trial’s effect on stress reduction (i.e., a group × time interaction). We estimated a pre-post correlation of .60 and a two-tailed Type I error rate of .05. To achieve 80% power, G*Power calculated a total of *N* = 147 participants needed to test the primary study aims using omnibus tests [33]. Treatment adherence and participant retention were encouraged in the following ways: Participants received standardized study reminders, individualized check-ins from a study manager, and cash incentives. Specifically, participants were contacted by an unblinded study manager during days 3 and 9 of the intervention to answer training-specific questions, address difficulties, and encourage program adherence. Participants also received bonus compensation for high adherence to the study protocol.

### Intervention programs

Participants were randomly assigned to one of three 2-week smartphone-based training programs (Monitor+Accept, Monitor Only, and Active Treatment Control). During the 2-week training period, participants completed daily 20-minute audio training sessions plus an additional 3–10 minutes of home practice. Lessons were completed in order and participants were not able to skip or repeat lessons. Each lesson trained specific techniques and provided opportunity for practice. To maximize experimental control, all three training programs were matched on attentional demand, length, structure, delivery tone and voice–all programs were delivered by the same female voice.

The training programs were developed in collaboration with mindfulness teacher Shinzen Young based on his Unified Mindfulness system [34]. The two mindfulness programs were designed to systematically parse mindfulness instruction into (1) attention monitoring and (2) acceptance. The content of each intervention is detailed in [10].

#### Monitor+Accept (MA) training program

The MA training program first taught foundational concentration skills which allowed participants to (1) monitor their present-moment physical experiences (e.g., physical sensations, muscle sensations, ongoing physiology, etc.) as well as body sensations related to emotions (e.g., anger, impatience, enthusiasm, anxiety, etc.) while (2) welcoming and accepting each of these experiences. Monitoring was explained in terms of two dimensions: (a) resolution–*discriminating* types of experiences (e.g., pleasant, unpleasant, neutral; physical vs. emotional) and (b) sensitivity–*detecting* subtle sensations (e.g., faint sensations related to pleasant activities and emotions). Acceptance was trained through three tangible strategies designed to embody the attitude of acceptance: (a) maintaining a state of global body relaxation, (b) mentally welcoming all physical and emotional body experiences, and (c) using a gentle, matter-of-fact tone of voice while labeling these experiences.

#### Monitor Only (MO) training program

The MO training program taught participants only to concentrate on and monitor present-moment physical and emotional body experiences (as described above), with no instruction regarding acceptance.

#### “My Time” control training program

The active treatment control training program was developed to be a useful stress management program that trained people to use common reappraisal and coping strategies, but without any training in mindfulness. It was developed to parallel the MA and MO training programs and to control for nonspecific effects of undergoing a training program (e.g., treatment expectancies, daily time and effort spent toward the goal of reducing stress). The “My Time” control program did not encourage focus on or acceptance of present-moment experiences. Instead, participants were instructed to reframe or reappraise past and anticipated events, and to analyze and try to solve personal problems.

### Measures

#### High sensitivity CRP assay

CRP was assayed from dried blood elutes using a high-sensitivity enzyme-linked immunosorbent assay (ELISA), using methods described in [35]. Initial validation of this method showed high correlation (*r* = .96) between serum and dried blood CRP in 94 paired samples [35]. Samples were run in duplicate in the Behavioral Immunology Lab at University of Pittsburgh within two years of sample collection. Each participant’s baseline and post-intervention blood samples were run on the same plate. Briefly, 3.2mm discs of samples, standards (Siemens Rheumatology Standards for CRP, at concentrations of 8.08, 4.04, 2.02, 1.01, 0.51, 0.25, 0.13, and 0mg/mL), and controls (Bio-Rad Liquichek Cardiac Markers, at concentrations of 0.37, 1.26, and 3.42mg/mL) were eluted in phosphate buffered saline (PBS) overnight, then loaded into a plate coated with 2 μg/mL mouse anti-human CRP antibody (Meridian Life Science #M86005M). CRP was captured using a 5ng/mL biotinylated detection antibody to CRP (Meridian Life Science #M01319M), 0.1ug/mL streptavidin-HRP (ThermoFisher #SNN2004). Chromogenic substrate (ACROS #327972000 + hydrogen peroxide) was then added and color development was stopped with 1M sulfuric acid after 28 minutes. Absorbance was read at 492nm and CRP concentrations were calculated using a four-parameter logistic regression fit to the standard curve. Average intra-assay coefficient of variation (CV) for high, medium, and low controls was less than 5% and average inter-assay CV was less than 15%.

#### Treatment adherence

The smartphone training application automatically timestamped the start and finish of each lesson in the 2-week training period. This electronic timestamp was used to calculate the total number of lessons completed for each individual.

#### Treatment expectancies

Participants completed an adapted 6-item Credibility/Expectancy Questionnaire [36] to assess their beliefs about the efficacy of the training program at post-intervention. Cognitive and emotional subscales were averaged to create an overall measure of treatment expectancies (Cronbach’s *α* = .95).

## Analyses overview

ANOVA and Maximum Likelihood Ratio Chi-Square tests were conducted with SPSS Statistics 25.0 software (IBM, Armonk, New York) to check for successful randomization using major demographics variables. Mixed Effects Linear Models (MLMs) were used to test for condition differences in CRP from baseline to post-intervention in Stata/SE 15.0 (StataCorp, College Station, Texas). MLMs allow all available data to be used in analyses while Repeated Measures ANOVAs use a list-wise subject deletion, removing subjects from analyses if any data is missing. This approach can lead to decreases in power and unequal sample sizes across conditions. In addition, MLMs are robust to violations of sphericity while RM ANOVAs can lead to inflated F-ratios if this assumption g. CRP concentrations were significantly positively skewed and thus log(10) transformation was used to normalize these data, as is commonly done in the literature. Primary analyses were conducted using log-transformed CRP values, and to visually depict changes in CRP, we report raw CRP in tables and figures. The repeated measure of time was modeled using identity covariance structure. In all MLM analyses the variables of interest (time, study condition) were modeled as fixed effects using maximum likelihood estimation. An alpha value of *p ≤* .05 was considered statistically significant.

## Results

### Preliminary analyses

**Fig 1:**
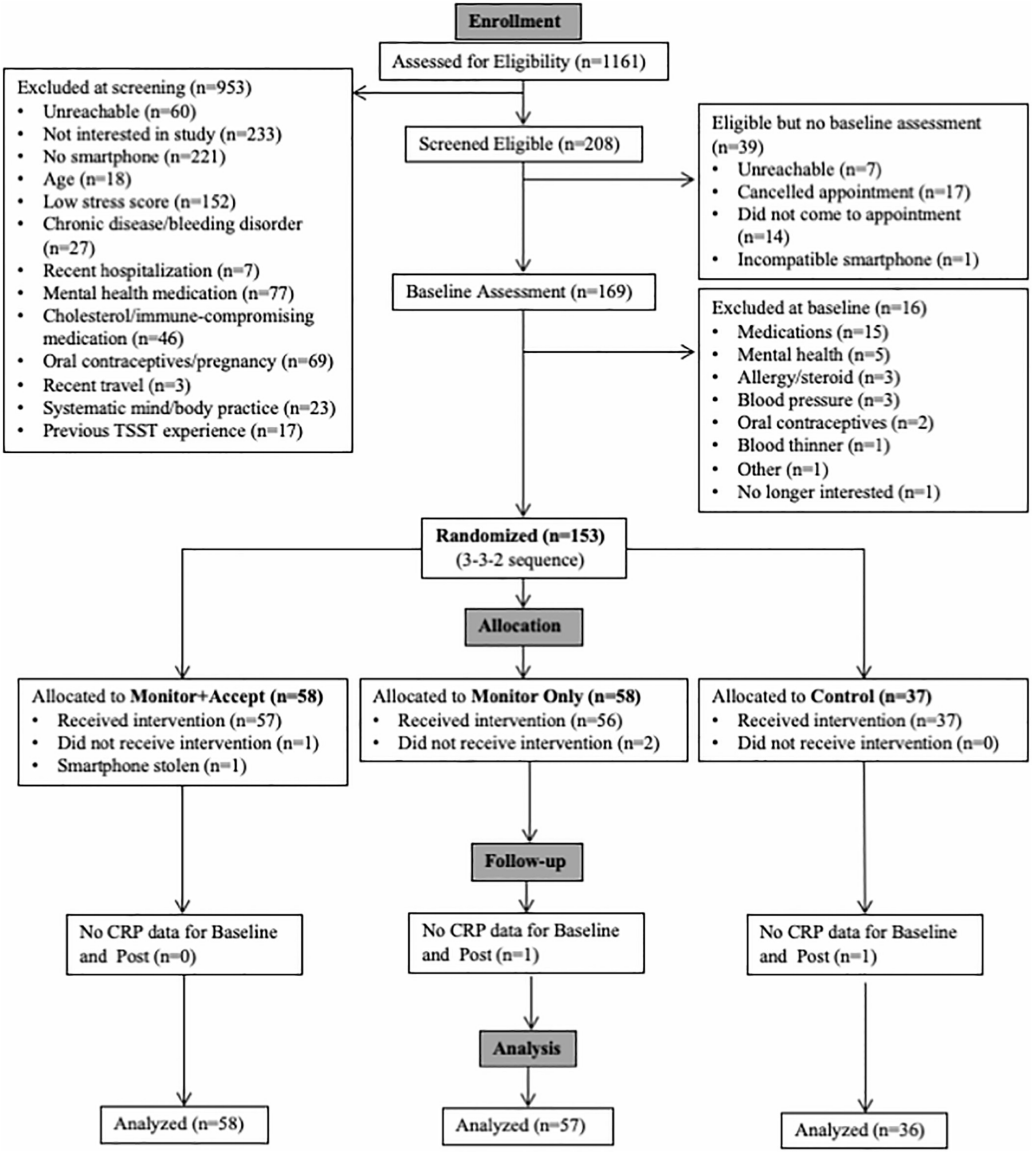
CONSORT flow charts.

Randomization was successful based on examination of major demographic variables. There were no baseline condition differences in age, gender, race, BMI, or education (see Table 1). A total of 149 participants (97% of the sample) returned to complete the post treatment assessment (See Fig 1 for CONSORT flow chart). Adherence to the program was excellent; the average number of sessions completed across the three groups was 13.5 (*SD* = 1.2) out of 14 possible sessions. There were no significant condition differences in the number of sessions completed (*F*(2,147) = .39, *p* = .68) or in treatment expectancies across the three groups (*F*(2,146) = 1.54, *p* = .22).

**Table 1:** Baseline characteristics for participants randomized in Study 1.

Characteristic	Full Sample (N = 153)	Control (N = 37)	MO (N = 58)	MA (N = 58)	Condition Difference
**Mean Age** years (SD)	32(14)	32(14)	33(13)	33(14)	*F*(2, 150) = .10, *p* = .91
**Sex**					
Female (%)	103(67%)	23(62%)	41(71%)	39(67%)	**χ**^*2*^(2) = 0.75, *p* = .69
Male (%)	50(33%)	14(38%)	17(29%)	19(33%)	
**Race**					
White (%)	81(53%)	25(68%)	28(48%)	28(48%)	**χ**^*2*^(8) = 14.49, *p* = .07
Black (%)	33(22%)	3(8%)	16(28%)	14(24%)	
Asian (%)	33(22%)	5(14%)	13(22%)	15(26%)	
Bi- or Multi-Racial (%)	5(3%)	3(8%)	1(2%)	1(2%)	
Other(%)	1(1%)	1(3%)			
**Education Level**					
GED (%)	3(2%)	1 (3%)	1(2%)	1(2%)	**χ**^*2*^(14) = 16.52, *p* = .28
High School Diploma (%)	20(13%)	1 (3%)	10(17%)	9(16%)	
Technical Training (%)	1(1%)			1(2%)	
Some college (%)	41(27%)	14(38%)	12(21%)	15(26%)	
Associate Degree (%)	10(7%)	4 (11%)	2(3%)	4(7%)	
Bachelor’s Degree (%)	48(31%)	12(32%)	21(36%)	15(26%)	
Master’s Degree (%)	26(17%)	5 (14%)	11(19%)	10(17%)	
MD, PhD, JD, PharmD (%)	4(3%)		1(2%)	3(5%)	
**Mean Baseline CRP in mg/mL** (SD) [95% CI]	1.43(1.76) [1.15–1.72]	1.30(1.50) [.78–1.80]	1.71(1.98) [1.18–2.23]	1.25(1.67) [.81–1.69]	*F*(2,147) = 1.10, *p* = .33

Note. *p* < .05, two-tailed

### Primary analyses

**Fig 2:**
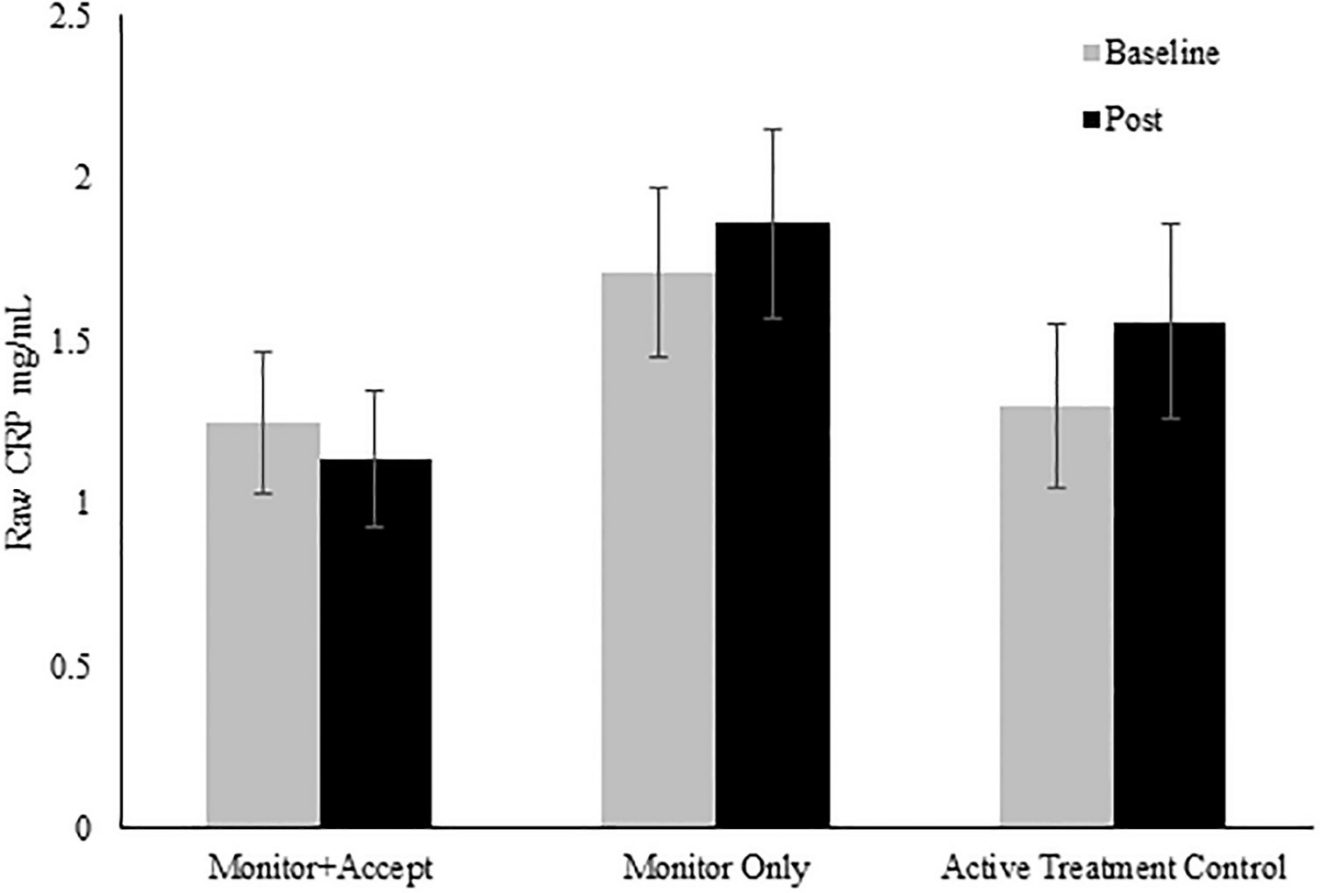
Study 1 raw CRP values before and after a 2-week smartphone intervention. Note: n = 151; Error bars refer to ±SE of the mean.

We hypothesized that the 2-week Monitor+Accept mindfulness program would significantly decrease circulating CRP in a sample of stressed community adults compared to the 2-week Monitor Only mindfulness training and active treatment control programs. Contrary to predictions, an MLM using all available data (n = 151) revealed no time effects (*χ*^2^(1) = 0.46, *p* = .50), condition effects (*χ*^2^(2) = 2.85, *p* = .24), or time x condition effects (*χ*^2^(2) = 1.17, *p* = .56; see Fig 2) on log-transformed CRP (see Table 2 for CRP means, SEs, and Cohen’s *d*).

**Table 2:** Study 1 raw and log-transformed CRP values.

**Raw CRP (mg/mL)**			
	Baseline *Mean (SE)*	Post *Mean (SE)*	*d*
**Monitor+Accept**	1.25 (.22)	1.14 (.21)	.10
**Monitor Only**	1.71 (.26)	1.86 (.29)	-.11
**No Treatment Control**	1.30 (.25)	1.56 (.30)	-.23
**Log CRP +1**			
	*Baseline* *Mean (SE)*	*Post* *Mean (SE)*	*d*
**Monitor+Accept**	.28 (.03)	.26 (.03)	.13
**Monitor Only**	.35 (.03)	.36 (.04)	-.06
**No Treatment Control**	.30 (.04)	.33 (.04)	-.19

Note: *d* = Cohen’s *d* effect size estimate.

## Study 1 Discussion

Study 1 examined whether a lower dose 2-week mindfulness training program delivered via a smartphone application decreased circulating CRP in a sample of stressed community adults compared to a 2-week mindfulness training program that did not train acceptance skills and an active treatment control program. Contrary to predictions, results showed that a 2-week mindfulness training program did not significantly reduce CRP at the group level. However, it is possible that reductions in circulating inflammation may occur after higher doses of mindfulness training. Study 2 used the standard 8-week MBSR course to examine whether mindfulness training reduces circulating levels of CRP.

## Study 2: 8-week MBSR course

This study was preregistered with Clinical Trials identifier NCT02502227. This manuscript describes changes in CRP after an 8-week MBSR course. All outcome measures reported here were not analyzed until data collection was complete.

## Method

### Participants

A total of 137 stressed community adults (67% women; *M*_*age*_ = 38, *SD* = 13) enrolled in the study. The majority of participants were White (66%), followed by Black (15%), Asian (10%), and Bi- or Multi-Racial (8%). Data collection occurred between August 2015 and November 2016. Sample size was determined based on power calculations conducted for daily stress and stress reactivity—the primary outcomes of the larger trial. Given that previous 8-week mindfulness interventions have demonstrated medium-large effect sizes for both daily stress and stress-reactivity outcomes relative to no treatment control groups (~η2 = 0.06–0.18), we conservatively estimated a small effect size (η2 = 0.02) for comparing the two active mindfulness training programs on changes in stress (i.e., a group × time interaction). We estimated a pre-post correlation of .60 and a two-tailed Type I error rate of .05. To achieve 90% power, G*Power calculated a total of *N* = 120 participants needed to test the primary study aims using omnibus tests. Participant retention and adherence was encouraged through reminder phone calls, text messages, and monetary incentives, including bonus compensation for high adherence to the study protocol. Participants assigned to the no treatment control group completed assessments at baseline and post-intervention (compliance was encouraged through check-in phone calls).

### Intervention programs

This 3-arm RCT was run in three cohorts. Each cohort was composed of 40–55 participants with intervention class sizes ranging between 15–22 participants. Two certified MBSR instructors taught both mindfulness courses, with instructors counterbalanced across cohorts. For each cohort, participants were randomly assigned to one of three study conditions: an 8-week MBSR course that explicitly trained both Monitor+Accept skills (MA), an 8-week modified MBSR course that explicitly trained Monitor Only skills (MO), or a no treatment control condition (NT).

Briefly, MBSR is a group-based program that consists of 8 weekly 2.5–3 hour-long in-person sessions, 1 day-long retreat, and approximately 45-minutes of daily home practice. During each group session, an MBSR instructor guides meditations and engages participants in discussions relevant to mindfulness in daily life. During the sixth week of the MBSR intervention participants attend a seven-hour retreat focused on integrating and elaborating on the mindfulness skills learned throughout the course. Finally, participants are asked to complete 45-minutes of guided meditation home practice and informal mindfulness exercises six days per week during the 8-week course.

#### Monitor+Accept (MA) MBSR course

The MA program refers to the standard MBSR course, with the exception that MA classes were 2 hours. During each group session, an MBSR instructor led guided mindfulness meditations intended to foster the ability to intentionally monitor present-moment experiences (pleasant, unpleasant, and neutral), and to cultivate an open, accepting, and non-judgmental attitude towards such experiences. Participants were asked to notice present thoughts, feelings, and body sensations and were invited to allow these experiences to be, without trying to change or improve them. Sessions included in-class activities and discussions about home practice, as well as group discussions about specific mindfulness skills and how to apply these skills to everyday life. The aim of these practices was to help individuals recognize and non-judgmentally monitor their unhelpful patterns of reacting to stress, and, over time, refine their ability to respond to stress more flexibly.

#### Monitor Only (MO) MBSR course

The MO program was adapted from the standard MBSR curriculum by co-author CG in consultation with a former senior teacher at the UMass Center for Mindfulness and utilizing meditation literature. The MO adaptations of the MBSR program consisted primarily of (1) changes in language, and (2) emphasis on concentration practices, specifically regarding body sensations. Changes in language included avoiding use of the words: allow, accept, acceptance, being with, letting go of judgment, non-judgment, non-interference, and instead referring to: direct perception, observe, monitor. The MO program emphasized focusing attention and returning to the focus object when becoming aware of distractions. One such practice was to anchor attention to breath, body sensations, or sound to return to the present moment. The MO program did not include some of the meditative practices that are typically included in standard MBSR courses, such as choiceless awareness and lovingkindness meditation, because these practices tend to foster attitudes of openness and acceptance. Stress was discussed in the MO program, and participants were guided to focus their attention on body and breath sensations as a resource for coping before, during, and or/after stressful events.

An MO curriculum guide was used by both teachers that included learning intentions, themes, session activities, and facilitator intentions. The curriculum guide included specific reminders regarding language and focus on concentration and monitoring practices. Teachers met prior to each session to review the curriculum.

#### No treatment (NT)

Participants assigned to the no treatment control group completed assessments on the same schedule as intervention participants (at baseline and post-intervention).

### Measures

#### High sensitivity C-Reactive Protein assay

CRP was assayed using the same procedures as in Study 1. Average intra-assay coefficient of variation (CV) for high, medium, and low controls was less than 10%, and average inter-assay CV was less than 15%.

#### Intervention and home practice adherence

Attendance was recorded by staff at each of the eight classes plus the day-long retreat and used to calculate the total number of classes attended for each participant. Home practice audio recordings were distributed each day using Qualtrics links that automatically recorded the duration of time each link was open. Durations longer than each audio recording were considered outliers, and were replaced with the actual duration of the audio recording (45 minutes). We calculated the total amount of home practice by summing daily time spent doing home practice during the eight weeks of the intervention.

#### Treatment expectancies

To evaluate whether both training programs produced equivalent perceived treatment benefits, participants completed an adapted 6-item Credibility/Expectancy Questionnaire [36] at the end of class on Weeks 1, 4, and 8. Responses from these three time points were averaged to create an overall measure of positive and negative expectancies (Cronbach’s *α* = .91, .92, and .91 at Weeks 1, 4, and 8).

## Analyses overview

As in Study 1, ANOVAs and Maximum Likelihood Ratio Chi-Square tests were conducted to check for successful randomization based on major demographic variables using SPSS software, and MLMs were used to test for condition differences in log-transformed CRP from baseline to post intervention using Stata software. Again, primary analyses were conducted using log-transformed CRP values, and raw CRP concentrations are reported in tables and figures. An alpha value of *p ≤* .05 was considered statistically significant.

## Results

### Preliminary analyses

Randomization was successful based on examining major demographic variables. There were no condition differences at baseline in age, gender, race, BMI, and education (see Table 3). A total of 125 participants (91% of the sample) returned to complete the post treatment assessment (See Fig 1B for CONSORT flow chart). Of the 107 participants assigned to one of the two active study interventions, 98 completed the intervention (92%). Class attendance was high and there were no differences in the number of classes attended between the two intervention groups (*F*(1,96) = 0.00, *p* = .98). On average, participants attended 7.44 of the 9 possible classes (8 weekly classes plus the day-long retreat). Moreover, 81 of 98 participants (83%) attended at least 6 of the 9 possible classes and only 9 participants (9%) attended fewer than half of the classes. MA participants tended to practice at home more than MO participants over the course of the 8-week intervention (*M*_diff_ = 2 hours and 51 minutes), but home practice did not significantly differ between the two mindfulness groups (*F*(1,96) = 2.97, *p* = .09). On average, participants across both mindfulness courses logged 12 hours and 14 minutes (out of 36 possible hours) of home practice during the 8-week intervention period.

**Table 3:** Baseline characteristics for participants randomized in Study 2.

Characteristic	Full Sample (N = 137)	Control (N = 30)	Monitor Only (N = 53)	Monitor + Accept (N = 54)	Condition Difference
**Mean Age** years (SD)	38(13)	41(13)	38(13)	36(14)	*F*(2,133) = 1.25, *p* = .29
**Sex**					
Female (%)	92(67%)	22(73%)	36(68%)	34(63%)	**χ**^*2*^(2) = 0.96, *p* = .62
Male (%)	45(33%)	8(27%)	17(32%)	20(37%)	
**Race**					
White (%)	91(66%)	22(73%)	36(68%)	33(61%)	**χ**^*2*^(8) = 8.65, *p* = .37
Black (%)	21(15%)	4(13%)	7(13%)	10(19%)	
Asian (%)	14(10%)	1(3%)	6(11%)	7(13%)	
Bi- or Multi-Racial	6(4%)	3(10%)	1(2%)	2(4%)	
Other	5(4%)		3(6%)	2(4%)	
**Education Level**					
No high school diploma (%)	1(1%)		1(2%)		**χ**^*2*^(16) = 13.01, *p* = .67
GED (%)	2(2%)		1(2%)	1(2%)	
High School Diploma (%)	10(7%)	3(10%)	3(6%)	4(7%)	
Technical Training (%)	2(2%)	1(3%)	1(2%)		
Some college (%)	18(13%)	5(17%)	4(8%)	9(17%)	
Associate Degree (%)	10(7%)	2(7%)	5(9%)	3(6%)	
Bachelor’s Degree (%)	41(30%)	5(17%)	17(32%)	19(35%)	
Master’s Degree (%)	40(29%)	11(37%)	17(32%)	12(22%)	
MD, PhD, JD, PharmD (%)	13(10%)	3(10%)	4(8%)	6(11%)	
**Mean Baseline raw CRP in mg/mL** (SD) [95% CI]	1.21 (1.59) [.94, 1.48]	1.05 (1.55) [.47,1.63]	1.16 (1.10) [.85, 1.46]	1.36 (1.97) [.82, 1.89]	*F*(2, 135) = .41, *p* = .665

Note. *p* < .05, two-tailed

### Primary analyses

**Fig 3:**
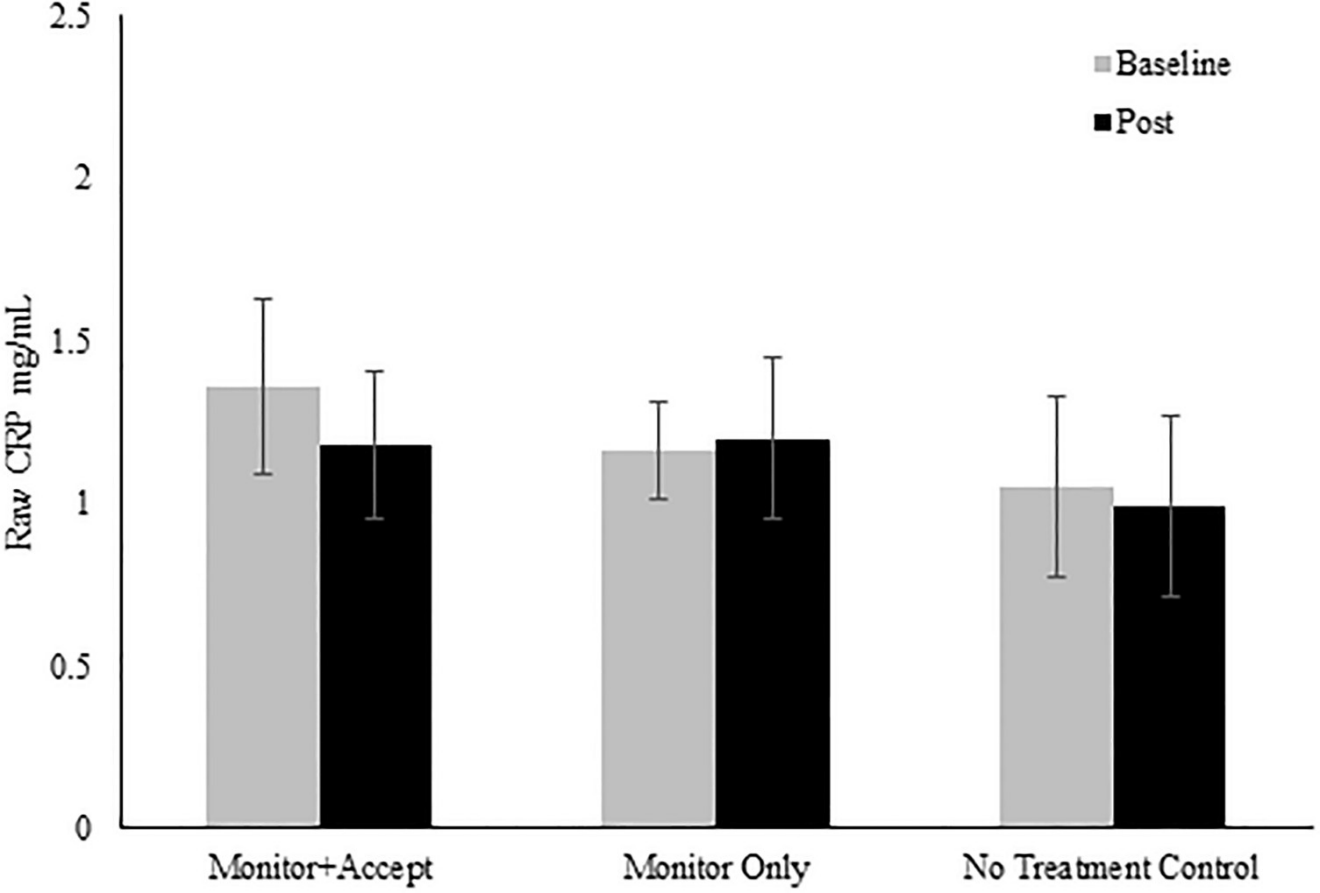
Raw CRP values before and after an 8-week MBSR course. Note: n = 133; Error bars refer to ±SE of the mean.

We hypothesized that MA training would lower circulating CRP levels compared to MO and the NT control group. Contrary to predictions, an MLM using all available data (n = 133) revealed no main effect of time across study conditions (*χ*^2^(1) = 1.19, *p* = .28), no main effect of condition (*χ*^2^(2) = 4.90, *p* = .09), and no time x condition interaction on log CRP (*χ*^2^(2) = 2.76, *p* = .25; see Fig 3 and Table 4 for raw and log-transformed CRP means, SEs, and Cohen’s *d*). Thus, neither 8-week MBSR program significantly affected circulating levels of CRP in a sample of stressed community adults, compared to the no treatment control group.

**Table 4:** Study 2 raw and log-transformed CRP values.

**Raw CRP (mg/mL)**			
	*Baseline* *Mean (SE)*	*Post* *Mean (SE)*	*d*
**Monitor+Accept**	1.36 (.27)	1.18 (.23)	.14
**Monitor Only**	1.16 (.15)	1.20 (.25)	-.04
**No Treatment Control**	1.05 (.28)	.99 (.28)	.06
**Log CRP +1**			
	*Baseline* *Mean (SE)*	*Post* *Mean (SE)*	*d*
**Monitor+Accept**	.12 (.04)	.09 (.04)	.16
**Monitor Only**	.14 (.04)	.11 (.05)	.14
**No Treatment Control**	.06 (.06)	.05 (.06)	.05

Note: *d* = Cohen’s *d* effect size estimate.

## Study 2 Discussion

Study 2 sought to examine whether an 8-week mindfulness intervention would reduce circulating CRP in stressed community adults, and whether training in acceptance skills was necessary for these effects. Specifically, we compared an MBSR program that trained both attention monitoring and acceptance skills to an MBSR program without acceptance training and a no treatment control group. Contrary to predictions, but mirroring Study 1, there were no significant changes in CRP from pre- to post-intervention and no condition differences.

## Exploratory subgroup analyses: Studies 1 & 2

Results from Studies 1 & 2 are consistent with previous mindfulness intervention RCTs that find no changes in systemic inflammation in healthy adult populations [37]. One possibility is that mindfulness interventions are only effective for reducing CRP in populations at higher risk for elevated inflammation. Indeed, mindfulness interventions have been shown to decrease CRP among overweight and older populations [20,21]–two groups known to show heightened levels of circulating markers of inflammation [38,39]. Therefore, it is possible that mindfulness interventions are only effective among groups that have heightened inflammation, and that floor effects prevented us from observing reductions in CRP in the full sample of stressed but healthy adults. To explore this prediction, we conducted subgroup analyses to examine whether our mindfulness interventions were more effective among overweight and obese individuals and those of older age. Since a small number of participants in each study met the criteria for overweight/obese or older age, we combined data from Studies 1 and 2 to maximize power for these exploratory analyses.

In the combined sample, higher BMI and older age were both significantly correlated with higher baseline CRP concentration (BMI: *r* = .471, *p*<.0005; age: *r* = .148, *p* = .013). Thus, exploratory analyses were conducted in the subgroup of participants classified by the CDC as overweight or obese (subgroup: BMI ≥ 25; n = 128) and in the subgroup of participants in the highest quartile of age within this (combined) sample (subgroup: age ≥ 45 years; n = 74). As would be expected, baseline CRP was significantly higher in overweight vs. normal weight (BMI ≥ 25: *M* = 1.97, *SE* = .14; BMI<25: *M* = .73, *SE* = .12; *F*(1,279) = 46.17, *p*<.0005) and older vs. younger subgroups (age ≥ 45: *M* = 1.74, *SE* = .19 ; age<45: *M* = 1.14, *SE* = .11; *F*(1,280) = 7.25, *p* = .008). We conducted two separate MLM analyses controlling for study number (whether the subject had participated in Study 1 or Study 2): One testing for time x condition differences in CRP for overweight/obese adults and one testing for time x condition differences among older adults.

In the subsample of overweight or obese participants from Studies 1 and 2 (n = 128), a MLM revealed a trend in the time × condition interaction for log-transformed CRP (*χ*^2^(2) = 5.29, *p* = .071). Both MA- and MO-trained participants tended to show decreases in CRP from baseline to post-intervention (MA mean change = -0.16, *p* = .095; MO mean change = -0.13, *p* = .145) whereas control participants tended to show increases in CRP (control mean change = 0.15, *p* = .17; Table 5). Both MA and MO participants showed significantly greater decreases in CRP compared to control (MA vs. control: *χ*^2^(1) = 4.51, *p* = .034; MO vs. control: *χ*^2^(1) = 3.88, *p* = .049), whereas MA and MO participants did not differ (*χ*^2^(1) = 0.07, *p* = .79). MO participants showed marginally greater decreases in CRP compared to control participants (*χ*^2^(1) = 3.61, *p* = .058).

**Table 5:** Exploratory subgroup analyses in Study 1 & 2 combined subsamples.

Outcome	Monitor+Accept^a^			Monitor Only^b^			Control^c^			Time x Condition Difference
	*Pre*	*Post*	*d*	*Pre*	*Post*	*d*	*Pre*	*Post*	*d*	
**Subgroup:** **BMI**≥**25**										
Log CRP + 1	1.06(.07)	0.99(.07)	.28	1.11(.06)	1.05(.06)	.25	1.04(.08)	1.10(.08)	-.24	***χ***^***2***^**(2) = 5.06**+
Raw CRP (mg/mL)	2.05(.31)	1.76(.31)	.27	2.01(.28)	2.03(.28)	-.02	1.88(.36)	2.09(.37)	-.20	*χ*^*2*^(2) = 3.51
**Subgroup:** **Age**≥**45**										
Log CRP + 1	.88 (.10)	.82(.11)	.19	1.14(.11)	1.10(.11)	.12	0.68(.14)	.87(.14)	-.57	***χ***^***2***^(2) = 7.26*
Raw CRP (mg/mL)	1.47(.38)	1.25(.38)	.19	2.17(.38)	2.38(.39)	-.20	1.49(.49)	2.12(.49)	-.54	***χ***^***2***^**(2) = 5.39**+

Note: Data are reported as means (SE) adjusted for study number.

^a^N = 43 for BMI subgroup analysis; N = 29 for Age subgroup analysis.

^b^N = 54 for BMI subgroup analysis; N = 28 for Age subgroup analysis.

^c^N = 31 for BMI subgroup analysis; N = 18 for Age subgroup analysis.

^+^p<.08 is considered marginally significant

*p<.05 is considered statistically significant

In the subsample of midlife and older participants (age ≥ 45 years) from Studies 1 and 2 (n = 74), an MLM revealed a significant time × condition interaction for log-transformed CRP (*χ*^2^(2) = 7.53, *p* = .02). This interaction was driven by significant increases in CRP among control participants (control mean change = 0.46, *p* = .013), whereas CRP did not significantly change from baseline to post-intervention among MA- and MO-trained participants (MA mean change = -0.13, *p* = .38; MO mean change = -0.11, *p* = .45; Table 5). Both MA and MO participants showed significantly greater decreases in CRP compared to control (MA vs. control: *χ*^2^(1) = 6.25, *p* = .012; MO vs. control: *χ*^2^(1) = 5.83, *p* = .016), whereas MA and MO participants did not differ (*χ*^2^(1) = 0.01, *p* = .925).

## General discussion

Mindfulness interventions have become increasingly popular, and well-controlled RCTs show that mindfulness training may have benefits for mental and physical health outcomes [15]. Despite this, the available evidence is mixed on whether mindfulness interventions reduce markers of systemic low-grade inflammation, such as CRP. We addressed this question with data from two pre-registered RCTs–a 2-week smartphone mindfulness program and an in-person group-based 8-week MBSR course–which examined whether mindfulness training reduced circulating levels of inflammation in stressed community adults. Given research showing that stress is a risk factor for elevated inflammation [6] and that mindfulness training can significantly reduce psychological and physiological stress responding [7,10], we expected that training both attention monitoring and acceptance skills would reduce stress-related elevations in circulating CRP. Contrary to expectations, but in line with some evidence from other RCTs [13], mindfulness training did not significantly change circulating levels of CRP. Since our results showed no reductions in CRP after a mindfulness intervention, we decided to examine whether individuals with additional risk factors for inflammation (middle-age and BMI of 25 or greater) would derive short-term inflammatory benefits from a mindfulness intervention. These exploratory subgroup analyses provide some initial evidence that mindfulness interventions may reduce CRP in populations at higher risk for heightened inflammation—stressed adults who are overweight or who are 45 and older.

Overall, the present work has a number of strengths that are worth highlighting. First, by testing the hypothesis that certain components of mindfulness lead to specific health-related improvements, we are moving towards a better understanding of how mindfulness interventions work, particularly regarding the training components that drive health-related benefits. Second, both studies included two versions of mindfulness training plus a control condition, thus providing a stringent test of the effectiveness of mindfulness interventions. In particular, the 2-week smartphone intervention included a well-matched active treatment control program that controlled for non-specific factors such as enrollment in a stress reduction course. This is a significant strength given that to date only a small percentage of RCTs have compared mindfulness interventions to active treatment controls rather than to wait-list or no treatment control conditions [40]. Third, we present results from two RCTs that used different delivery approaches and mindfulness doses. Although most of the research has focused on examining the effects of 8-week MBSR courses, as mindfulness continues to grow in popularity, research needs to examine the effectiveness of alternative, more cost-effective delivery methods that are scalable and more accessible to a wider population (e.g., those with time and financial restrictions). Fourth, for the 8-week MBSR intervention we recorded the amount of time spent in home practice. Given that home practice is a key component of the MBSR program, having a proxy for home practice engagement lowers concerns that participants enrolled in the interventions did not engage with the material outside of the weekly classes.

Recent findings, including our own, support the view that learning acceptance skills is important for the physiological and psychological benefits of mindfulness intervention on markers of stress [10,28]. The present findings suggest that this does not extend to circulating markers of inflammation, which did not differ between Monitor+Accept and Monitor Only interventions. We offer two explanations for these findings. First, Lindsay et al. (2018) observed reduced cortisol and blood pressure reactivity for the Monitor+Accept group compared to Monitor Only and active treatment control group in response to an acute social stressor task; it is therefore possible that mindfulness interventions are more effective at down-regulating physiological responses to acute challenges. Second, participants in both studies had relatively low levels of baseline inflammation (Study 1 CRP: *M* = 1.43, *SD* = 1.76; Study 2 CRP: *M* = 1.21, *SD* = 1.59). Based on previous research showing a relation between stress and inflammation [6], we recruited stressed community adults under the assumption that these individuals would have higher levels of inflammation compared to community adults who did not report high to moderate levels of stress. However, these participants were relatively healthy in terms of their inflammatory profile. Thus, we may have encountered a floor effect with limited room for this measure to change in response to intervention. As one preliminary method to examine this possibility and explore whether there are subgroups of individuals who may derive greater short term benefit from mindfulness intervention, we conducted exploratory analyses examining whether mindfulness training was more effective for subgroups of individuals who were overweight/obese or 45 years or older (both groups that demonstrated heightened baseline CRP compared to their younger and lower BMI counterparts). These exploratory analyses tentatively suggest that both mindfulness interventions may be effective at reducing CRP compared to controls among these subgroups.

Results from the present work contribute to our understanding of how and under what conditions mindfulness interventions may be most effective. First, these results are consistent with previous findings showing that mindfulness interventions have little inflammatory benefit among more healthy populations [19], although it is possible that longer, more intensive interventions may be needed to observe changes in inflammation. Second, based on the present work, stressed individuals who are at increased risk for inflammation by virtue of being overweight/obese or of older age may potentially derive the most health benefits from mindfulness interventions. Third, a recent review of the effectiveness of mind-body interventions on inflammatory biology reported that mind-body interventions that include physical activity (e.g., yoga, Tai Chi, Qigong) more consistently report decreases in CRP than mindfulness interventions [37]. Therefore, it is possible (and worthy of future research) that interventions intended to influence the processes that lead to changes in CRP may need to focus more on physical activity in order to increase the likelihood of observing significant reductions.

A limitation of the present work is that participants in both studies had relatively low levels of CRP at baseline which could have prevented us from observing reductions after the mindfulness interventions. Nonetheless, these findings are consistent with previous research showing that mindfulness training does not improve markers of inflammation among healthy individuals [17–19]. Additionally, the exploratory findings were conducted by combining samples across study 1 and study 2. A clear limitation of these results is that the interventions had different lengths and delivery methods which might affect circulating levels of CRP through different underlying processes. Future research might consider examining whether mindfulness interventions can reduce systemic inflammation in samples with heightened low-grade inflammation [20] or with multiple risk factors for heightened inflammation.

## Conclusion

In conclusion, data from two pre-registered RCTs did not support the hypothesis that mindfulness interventions significantly decrease circulating levels of CRP, a clinically-relevant marker of systemic inflammation in stressed community adults. Future research is warranted to examine the possibility that stressed adults with high BMI and stressed midlife and older adults–individuals who are at increased risk for inflammatory pathophysiology–may experience greater health benefit from mindfulness training than their stressed counterparts who are at lower risk of systemic inflammation.

## Supporting information

S1 File Study 1 and Study 2 CRP combined data BMI_ = 25 082018. (DTA)

S2 File Study 1 CRP data 082018. (DTA)

S3 File Study 2_CRP data 082018. (DTA)

S4 File Villalba CONSORT checklist. (DOC)

S5 File Study 1 and Study 2 CRP combined data 082018. (DTA)

S6 File Study 1 and Study 2 combined data age_ = 45 082018. (DTA)

S7 File Villalba Study 1_IRB Proposal. (DOCX)

S8 File Villalba Study 2_IRB Proposal. (DOCX)
