# Supplementary material for: Mindfulness training and systemic low-grade inflammation in stressed community adults: Evidence from two randomized controlled trials
Source: PLoS One. 2019 Jul 11;14(7):e0219120. doi: 10.1371/journal.pone.0219120 (PMC6622480; doi:10.1371/journal.pone.0219120)
Supplement: S8 File — (DOCX) [file pone.0219120.s008.docx]

| 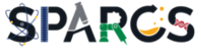 |
| --- |

| \| \| Date: Monday, September 10, 2018 11:08:47 AM \|  \|  \| \| --- \| --- \| --- \| \|  \|  \|  \|  \|  \| \| View: CMU-SF: Basic Information \| \| --- \| \| \| --- \| --- \| --- \| \| \| --- \| --- \| --- \| --- \| --- \| --- \| --- \| --- \| --- \| --- \| |
| --- | --- | --- | --- | --- | --- | --- | --- | --- | --- | --- |
| Basic Information [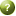](https://www.cmu.edu/research-office/sparcs/helptext/irb-help-text.pdf#nameddest=basicinfo)   1. * **Title of Study:** 8-week Attention Training and Stress Reduction 2. * **Brief description:** The purpose of the study is to test how training attention can impact stress. The study activities will take place over 10 weeks and will consist of an initial in-person session, followed by 3 days of surveys on a smartphone, an 8-week training program or waiting period, an additional 3 days of surveys on a smartphone, and a final in-person session. 3. * **Principal investigator:** John Creswell  **Title:** Associate Professor **Department:** PSYCHOLOGY |
| \| \|  \| \| View: CMU-SF: Study Team Members \| \| --- \| \| \| --- \| --- \| --- \| \| \| --- \| --- \| --- \| --- \| |
| Study Team Members      [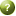](https://www.cmu.edu/research-office/sparcs/helptext/irb-help-text.pdf#nameddest=studymembers)  For the purpose of this submission, study team members to be listed are CO-Is and Study Faculty Advisors.  We recognize that other team members may be involved with the work with the PI being responsible for appropriate training.   1. **Identify each Co-Investigator and Faculty Advisor.  These should be individuals involved in the design, conduct or reporting of the research. Who should be included as a Co-I is at the discretion of the PI**[**(see OHRP Guidance on “who are investigators?”)**](http://www.hhs.gov/ohrp/regulations-and-policy/guidance/faq/investigator-responsibilities/)**.  Faculty Advisors must be listed for students serving as a PI.  *All team members who interact with participants or who have access to identifiable research data, whether listed here or not, must complete CITI training on Human Subjects Research.***  \| \| Name \| Roles \| Involved in Consent \| E-mail \| Phone \| Title \| Department \| \| --- \| --- \| --- \| --- \| --- \| --- \| --- \| \| Emily Lindsay \| CO-I \| yes \| elindsay@andrew.cmu.edu \| +1 (412) 268-8113 \| Job Mgmt Student Job Profile \| PSYCHOLOGY \| \| Hayley Rahl \| CO-I \| yes \| hrahl@andrew.cmu.edu \| +1 (412) 268-8761 \| Research Associate II \| PSYCHOLOGY \| \| \| --- \| --- \| --- \| --- \| --- \| --- \| --- \| --- \| --- \| --- \| --- \| --- \| --- \| --- \| --- \| --- \| --- \| --- \| --- \| --- \| --- \| --- \| \|  \|  1. **Identify any additional persons from external institutions who are also involved in the design, conduct, or reporting of the research, but who were not available in the preceding selector.  NOTE: If this is a multi-site study requiring sIRB review at CMU, please include your CO-I’s from all participating sites:**  \| \|  \| First Name \| Last Name \| Email \| Institution \| SPARCS Account Requested \| \| --- \| --- \| --- \| --- \| --- \| --- \| \|  \| There are no items to display \| \| \| \| \| \| \| --- \| --- \| --- \| --- \| --- \| --- \| --- \| --- \| --- \| --- \| --- \| --- \| --- \| \|  \|  1. * **Please briefly describe the qualifications and responsibilities of each study team member in regards to the research.  Include the PI and all persons listed above.  Please list a few sentences for each study team member, describing responsibilities and relevant expertise. (*This question must be answered to satisfy a regulatory requirement.*)**   **Examples:   Andrew Carnegie will serve as PI on this project and will oversee all aspects of the research, from design through data analysis and publication of the results. Dr. Carnegie is a professor in the college of engineering who has 20 years of experience conducting research. His research interests include…   Dr. Mellon will serve as a Co-I on this project. He is a physician from the Bayside Healthcare System who will oversee the research design, specific to the medical needs of the subject population. Dr. Mellon has been treating and researching this subject population for 30 years.  Scotty Carnegie is a PhD candidate whose work will be overseen by the PI.  He will assist with the data collection and analysis.**  Dr. John David Creswell is the principal investigator of this study. He creates, directs and oversees all study activities. Dr. Creswell is an associate professor in the Department of Psychology in Dietrich College, who has had roughly 2 decades of experience conducting research. His research interests include self-regulation, stress and coping, mind-body relationships, behavioral interventions, meditation, research methods, the science of consciousness, and performance enhancement.   Emily Lindsay is a PhD student who is under the direction of Dr. Creswell. She is heavily involved in the design, conduct, and reporting of the study details and will be expected to complete aspects of analysis once all data is collected.   Hayley Rahl is Dr. Creswell's lab manager and is involved in all aspects of the study, including the design, conduct, and reporting of the research. |
| \| \|  \| \| View: CMU-SF: CITI Training \| \| --- \| \| \| --- \| --- \| --- \| \| \| --- \| --- \| --- \| --- \| |
| Study Team Training [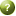](https://www.cmu.edu/research-office/sparcs/helptext/irb-help-text.pdf#nameddest=membertraining)    **CITI Training for Study Team Members**:   \| \| Name \| Training \| Uploaded Training Documentation \| \| --- \| --- \| --- \| \| Hayley Rahl \| \| Course \| Group \| Stage \| Completion Date \| Expiration Date \| \| --- \| --- \| --- \| --- \| --- \| \| Responsible Conduct of Research \| Social and Behavioral Responsible Conduct of Research Course \| Basic Course \| 9/1/2012 \|  \| \| Social & Behavioral Research - Basic/Refresher \| Social & Behavioral Research - Basic/Refresher \| Refresher Course \| 11/17/2015 \| 11/16/2018 \| \| Social & Behavioral Research - Basic/Refresher \| Social & Behavioral Research - Basic/Refresher \| Basic Course \| 12/17/2012 \| 12/16/2015 \| \|  \| \| Emily Lindsay \| \| Course \| Group \| Stage \| Completion Date \| Expiration Date \| \| --- \| --- \| --- \| --- \| --- \| \| Social & Behavioral Research - Basic/Refresher \| Social & Behavioral Research - Basic/Refresher \| Basic Course \| 7/25/2015 \| 7/24/2018 \| \|  \| \| \| --- \| --- \| --- \| --- \| --- \| --- \| --- \| --- \| --- \| --- \| --- \| --- \| --- \| --- \| --- \| --- \| --- \| --- \| --- \| --- \| --- \| --- \| --- \| --- \| --- \| --- \| --- \| --- \| --- \| --- \| --- \| --- \| --- \| --- \| --- \| --- \| --- \| --- \| --- \| --- \| \|  \|   **CITI Training for Principal Investigator:**   \| \| Course \| Group \| Stage \| Completion Date \| Expiration Date \| \| --- \| --- \| --- \| --- \| --- \| \| There are no items to display \| \| \| \| \| \| \| --- \| --- \| --- \| --- \| --- \| --- \| --- \| --- \| --- \| --- \| --- \| \|  \|   **Training Documentation for Principal Investigator (only if PI is external to CMU):** |
| \| \|  \| \| View: CMU-SF: Funding Sources (integrated with Grants) \| \| --- \| \| \| --- \| --- \| --- \| \| \| --- \| --- \| --- \| --- \| |
| Funding Sources [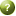](https://www.cmu.edu/research-office/sparcs/helptext/irb-help-text.pdf#nameddest=funding)   *** Is this funded research?**   **Yes** No  **Select SPARCS Funding Sources:**   \| \| Project ID \| Project Name \| Project State \| Project Type \| PI \| Sponsor Name \| \| --- \| --- \| --- \| --- \| --- \| --- \| \| There are no items to display \| \| \| \| \| \| \| \| --- \| --- \| --- \| --- \| --- \| --- \| --- \| --- \| --- \| --- \| --- \| --- \| --- \| \|  \|   **Select non-SPARCS Funding Sources**:   \| \|  \| Funding Source (from list) \| Funding Source (manual entry) \| SPEX ID \| Attachments \| Is Internal / CMU Department \| \| --- \| --- \| --- \| --- \| --- \| --- \| \| [View](https://sparcs.andrew.cmu.edu/irb/ResourceAdministration/Project/PrintSmartForms?Project=com.webridge.entity.Entity%5BOID%5B8809FBF1C9DBD547B44CE3C93A19FFC2%5D%5D&rootEntity=com.webridge.entity.Entity%5BOID%5B8809FBF1C9DBD547B44CE3C93A19FFC2%5D%5D&PrintBySection=False&PrintHeaderView=False&PrintHeaderInfo=True&PrintPageBreak=True&PrintLogo=True&showHiddenData=False&showCDTFormsAtEnd=True) \| NATIONAL INSTITUTES OF HEALTH (NIH) \|  \| A016939 \| [R21 Grant Application](https://sparcs.andrew.cmu.edu/irb/Doc/0/RJ1HF1SEQR1KL6JSOM7CRVT1F4/Creswell%20Summary%20Statement%20R21AT008493-01A1.pdf) \| no \| \| \| --- \| --- \| --- \| --- \| --- \| --- \| --- \| --- \| --- \| --- \| --- \| --- \| --- \| \|  \| |
| \| \|  \| \| View: CMU-SF: Review Type Requested \| \| --- \| \| \| --- \| --- \| --- \| \| \| --- \| --- \| --- \| --- \| |
| Review Type Requested [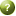](https://www.cmu.edu/research-office/sparcs/helptext/irb-help-text.pdf#nameddest=reviewtype)  This choice will determine what type of review and approval your submission will receive as well as the type of questions which will follow this page. Requesting the correct type of review is important to avoid delays in the review. If you are unsure about which type of request is appropriate please contact the IRB Office at [irb-review@andrew.cmu.edu](mailto:irb-review@andrew.cmu.edu).     1. * **What type of review are you requesting?** Non-Exempt (Expedited/Full Board) |
| \| \|  \| \| View: CMU-SF: Study Scope \| \| --- \| \| \| --- \| --- \| --- \| \| \| --- \| --- \| --- \| --- \| |
| Study Scope [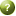](https://www.cmu.edu/research-office/sparcs/helptext/irb-help-text.pdf#nameddest=studyscope)   1. * **Does the study do any of the following:**    - Specify the use of an approved drug or biologic?    - Use an unapproved drug or biologic?    - Use a food or dietary supplement to diagnose, cure, treat, or mitigate a disease or condition?     Yes **No**   1. * **Does the study evaluate the safety or effectiveness of a device?**   Yes **No** 2. * **Provide, in lay terms, a summary of your proposed study:** This work tests the mechanisms of mindfulness training. We take the two components which are widely believed to describe mindfulness (monitoring and accepting present-moment experience) and we offer two predictions about how these active components affect attention and stress reactivity. Specifically, our account posits that (1) learning how to monitor present moment experience in mindfulness training interventions is sufficient to improve attentional control (which has been suggested in previous mindfulness training studies), but (2) learning how to both monitor and accept present moment experience is necessary for stress reduction. To test the two components we will separate the components into two distinct mindfulness training conditions that we will be administered over the course of 8 weeks via in-person training sessions. Attention training programs will include mindfulness meditation techniques as well as a no-treatment control condition. On participants’ personal smartphones, we will monitor psychological and neuroendocrine (cortisol, via saliva samples taken at signaled time points) stress responding before and after the intervention programs using Ecological Momentary Assessment (EMA) methods. After the post-intervention EMA collection, participants will return to the lab to complete questionnaires and task-based measures and to complete a laboratory performance challenge task (the Trier Social Stress Task (TSST), which we have previously administered in several studies). Physiological measures (heart rate, blood pressure, cortisol, etc.) will be used to assess stress responding in the laboratory. We will also collect blood spots at the baseline and post sessions for later quantification of inflammatory markers. 3. * **What is the purpose of the study (what is your research question) and how will the data collected be used?** The purpose of this study is to test the mechanisms of mindfulness training, evaluating whether it is monitoring or monitoring plus acceptance of present moment experience during mindfulness training that reduces stress. This project has the potential to identify how these putative psychological mechanisms of mindfulness training affect stress reduction outcomes. We predict that psychological and neuroendocrine stress levels will decrease among participants who receive traditional (monitoring and accepting) mindfulness training, as measured naturalistically from pre- to post-intervention using EMA methods, as well as during a post-intervention laboratory performance task. All collected data will be used to test for differences in stress-responding between study conditions and moderated by individual difference factors. Results will be submitted for publication in scientific journals and will be used to inform future studies. 4. * **For each activity/participant population, describe the research procedures:** During an initial pre-screening phone call, the study will be explained in greater depth and participants will complete a screening questionnaire consistent with the inclusion and exclusion criteria specified in inclusion/exclusion criteria section; and all participants must report a significant amount of stress in the past month as assessed by items from the Perceived Stress Scale (Cohen, Kamarck, & Mermelstein, 1983). Eligible participants will schedule their study start date, and visit the laboratory to provide informed consent, and complete baseline psychosocial questionnaires assessing thoughts, feelings, and individual differences (self-reported and task-based mindfulness, attention, stress, coping, affect, etc.). They will register to receive EMA notifications on their smartphones. We will inform participants that these EMA notifications will enable our research team to provide them with a report at the completion of their participation in the study that outlines their reported levels of attention, mood, and stress over the course of the study. Finally, we will collect blood spots from participants for later quantification of inflammatory markers.  Blood Spot collection procedures: Dried Blood Spot (DBS) assays will be used to measure markers of inflammation in this study. DBS collection is simple, fast, painless for most, and less invasive than venipuncture, but correlates highly with analyses from fresh blood collected via venipuncture. Details about this procedure are outlined here (http://www.jove.com/video/50973/dried-blood-spot-collection-health-biomarkers-to-maximize). Research staff will be trained in sample collection. First, they will complete Blood Borne Pathogen Training, which covers topics for handling and disposing of sharp lancets, personal protective equipment (disposable gloves, lab coats, long pants, closed shoes) and sanitizing, and how to respond to blood borne pathogen exposure. For sample collection, the participant will wash hands and warm them. The research assistant (wearing nitrile gloves and protective gear) will clean the selected finger twice with alcohol pads, then squeeze it to stimulate blood flow at the puncture site. The finger pad is punctured with a sterile safety lancet that is disposed in a sharps container. The finger is squeezed again until a blood droplet forms at the puncture site, which is then dropped onto collection paper. This procedure is repeated until several blood drops are collected. The puncture site is cleaned and bandaged. The collection paper is dried for 15-30 minutes, then inserted into a biohazard specimen bag and frozen until analysis. Finally, the blood collection area is cleaned and all waste is deposited into a biohazard waste bag.  After the baseline appointment, participants will complete several consecutive days of Ecological Momentary Assessment (EMA) on their smartphones. Participants will be prompted on their smartphone to complete brief (5-10 minutes at most) questionnaires assessing their thoughts and feelings (e.g., stress perceptions, mind wandering) (~six times per day). After this baseline EMA data collection, participants will begin their assigned attention training (participants in the no-treatment control condition will not complete attention training on these days, but may be asked to complete EMA questionnaires as the other conditions).  Participants will be randomly assigned to one of 3 arms: (1) the standard 8-week Mindfulness-Based Stress Reduction (MBSR) program, which emphasizes monitoring and acceptance training in each program training activity (Monitor + Accept Training Condition); (2) an adapted 8-week MBSR program, with all activities now focusing on monitoring training only (Monitor Only Condition); or (3) an assessment only No Treatment Control condition. The active programs will consist of guided meditation, slow stretching, and group interaction with a trained facilitator.   The MBSR classes will be held at the Center for Sustainable Landscapes at Phipps Conservatory and Botanical Gardens and will be videotaped with the videocamera positioned on the instructor. These videotapes will be used by research staff primarily for training purposes. Additionally, we aim to use these videos in two ways: first, blinded staff members will code these videos for the instructor's specific behaviors (e.g. instructor's use of acceptance language). Second, if participants miss their assigned class session, they will be given the opportunity to come to the lab to watch the video of the class(es) they missed.   These programs also contain a 6-8 hour day long program where participants integrate and practice all program activities in day long format.  After completion of the 8-week training programs, participants will complete several more days of Ecological Momentary Assessment on their smartphones, completing questionnaires as they did at baseline. Then, participants will be scheduled for a final study session in which they will arrive between the hours of 2-8pm (to control for natural diurnal variation in cortisol, which will be assessed at baseline and several times throughout the session with saliva samples). Participants will provide blood for post-intervention dried blood spot assays. Then, they will be fitted with a blood pressure cuff and sensors that monitor autonomic nervous system activity, which are described below (following paragraphs). Participants will complete the same questionnaires and task-based measures as at the baseline appointment. They will be asked to complete a performance challenge task (the TSST), and given instructions for that task; specifically, they will have to give a 5-minute speech detailing why they should be hired for a specific job position and convince evaluators (two study confederates) that they should receive a job offer. Then, participants will complete a brief attention training session (consistent with their assigned study condition; no treatment control participants will listen to a neutral-themed audio recorded story at this time, or sit quietly during the training period). We will again collect self-report and behavioral measures of participants’ thoughts and feelings before the performance task begins. Two evaluators (wearing white lab coats and equipped with clip boards) will enter and direct the actual performance tasks (lasting 10 minutes), which will be videotaped for facial affect coding. These videotapes will be reviewed by research assistants for inappropriate content and then hosted on a private, online repository (i.e. private youtube Channel) where paid crowd workers can help analyze video recording data. Workers will code the videotapes for behavioral markers of various dimensions (e.g. positive affect). The evaluators are trained to act in a nonaccepting manner toward the participant’s performance during these tasks. These exercises are an adapted version of the Trier Social Stress Test (Kirschbaum et al., 1993), which is a common laboratory challenge task that has been approved by the IRB and used in many behavioral studies, including several studies in our lab (e.g. Creswell et al., 2005, 2013). After completion of the tasks, the evaluators will exit and participants will complete final questionnaires and behavioral measures assessing post-task feelings, as well as providing additional saliva samples. Participants will then be thoroughly debriefed and thanked for their participation, and excused after receiving their compensation.  Physiological Activity measurement: The salivary and cardiac measures we are collecting in this study are non-invasive and present minimal risk to participants. If a participant reports any discomfort, we will immediately make adjustments in our physiological measurement to remedy them (e.g., adjust the sensors/cuff so that they are more comfortable or remove the sensors/cuff, if needed). Salivary cortisol will be assessed using a Salivette device (Sartstedt & Rommelsdorf, Germany), which contains a small cotton roll and sealed plastic container. At each assessment, participants will be asked to place the cotton roll under their tongue for three minutes, and then place the cotton roll in the corresponding labeled plastic container. Salivary cortisol will be collected at four different points during the experimental session: prior to hearing the instructions for the performance task, after the ten minute post-task rest period, after filling out the post-task questionnaires, and after the study debriefing. All Salivette devices will be frozen at -20º Celsius in a locked and secure laboratory freezer, and after all participants have completed the experiment the Salivettes will be placed over dry ice and mailed to a professional laboratory specializing in cortisol measurement. At this laboratory, cortisol will be measured using a high sensitivity salivary cortisol immunoassay kit (Salimetrics, State College, PA, USA, sensitivity <.007 ug/dL). Skin conductance responses (SCR) will be recorded from the volar surface of the distal phalanges on the index and middle fingers of the subjects’ non-dominant hand using two Ag/AgCl sensors with double sided-adhesive collars and an electrode paste (0.5M NaCl). Galvanic skin conductance responses will be measured using MindWare’s EDA 2.1 computer program that will detect any change in skin conductance greater than 0.5µ. We will calculate tonic skin conductance level (SCL), which has been used as a measure of sympathetic arousal over longer periods of time (i.e. minutes versus seconds) (Wallin, 1981). Because the use of soap and water can affect skin conductance, and the length of time passed since the last hand washing will vary for each participant, all participants will be required to wash their hands with a nonabrasive soap and water and thoroughly dry their hands prior to sensor placement. Ambient room temperature and time of day will also be controlled. Electrocardiograph (EKG) signals will be recorded using a standard lead II configuration (right arm, left ankle, with a right leg ground) with the left ankle having the positive lead. Impedance cardiograph (ZKG) signals will be recorded using Ag/AgCl spot sensor disks with adhesive collars in a tetrapolar sensor configuration (Qu, Zhang, Webster, & Tompkins, 1986). One current sensor is placed on the back of the neck, over the fourth cervical vertebra, and another current electrode is placed on the back, over the ninth thoracic vertebra. One voltage sensor is placed on the front of the neck, 4 cm above the clavicle, and another voltage sensor is placed over the sternum, at the fourth rib. Skin will be cleansed with an alcohol swab prior to sensor placement. A 4-mA alternating current at 11kHz will be passed through the two current sensors. Basal transthoracic impedance (Z0) and the first derivative of basal impedance (dZ/dt) will be recorded from the two voltage sensors (MindWare BioLabs Version 2.1, MindWare Technologies, Gahanna, OH). A lab computer will store this information. From the EKG, Z0, and dZ/dt signals, five measures will be calculated: a) pre-ejection period (PEP)- a measure of contractile force, b) cardiac output (CO)- liters of blood ejected from the left ventricle per minute, c) total peripheral resistance (TPR)- overall systemic vascular resistance calculated using the formula MAP/CO x 80 (see below for blood pressure measurement), d) heart rate (HR)- a rate-based measure of cardiac performance, and e) heart rate variability (HRV)- a measure of beat-to-beat variations in heart rate. Oscillometric blood pressure will be measured using an automatic sphygmomanometer (Dinamap Carescape V100, General Electric Company, GE, Finland). Mean arterial (MAP), systolic (SBP), and diastolic (DBP) blood pressure will be recorded. The cuff will be placed above the brachial artery of the participants’ non-dominant arm. Respiration rate and volume will be measured with a respiration belt placed around the participant’s torso (on the outside of their clothing). After being fitted with these sensors, the participant will remain in the same room for the entire experiment until the sensors are removed at the end of the experiment. 5. * **For each activity/participant population, indicate the location(s). Specify whether the participant will be engaged in person, remotely via the internet, etc.:** Eligible participants will schedule their study start date, and visit the laboratory to provide informed consent, and complete baseline psychosocial questionnaires assessing thoughts, feelings, and individual differences (self-reported and task-based mindfulness, attention, stress, coping, affect, etc.).  After the baseline appointment, participants will complete several consecutive days of Ecological Momentary Assessment (EMA) on their smartphones.  The MBSR classes will be held at the Center for Sustainable Landscapes at Phipps Conservatory and Botanical Gardens and will be videotaped with the videocamera positioned on the instructor.   After completion of the 8-week training programs, participants will complete several more days of Ecological Momentary Assessment on their smartphones, completing questionnaires as they did at baseline.  Then, participants will be scheduled for a final study session in which they will arrive at the laboratory between the hours of 2-8pm (to control for natural diurnal variation in cortisol, which will be assessed at baseline and several times throughout the session with saliva samples) 6. * **For each activity/participant population, describe the time required of the participant:** Initial pre-screening call: approximately 20 minutes   Baseline appointment: approximately 2 hours   Before Ecological Momentary Assessment (EMA) surveys: 5-10 minute surveys six times a day for three days before the intervention   Mindfulness-based Stress Reduction Classes: 2 hour class sessions for an 8-week duration and a 6-8 hour long retreat day where participants integrate and practice all program activities  After Ecological Momentary Assessment (EMA) surveys: 5-10 minute surveys six times a day for three days after the intervention   Post session appointment: approximately 3 hours 7. * **Who will be asked to participate?** Pittsburgh community members (age 18+) 8. * **Will questionnaires or surveys be used?**   **Yes** No   **If yes, please attach:**   \| \| Name \| \| --- \| \| [Baseline Questionnaire 1](https://sparcs.andrew.cmu.edu/irb/Doc/0/JIENCM92ALM438TKPNUOQVAIB4/Baseline%20Questionaire%201.docx) \| \| [Baseline Questionnaire 2](https://sparcs.andrew.cmu.edu/irb/Doc/0/GOG3MHNEPBKKF828HQM1FR2L0B/Baseline%20Questionaire%202.docx) \| \| [MBSR Class Eight Questionnaires](https://sparcs.andrew.cmu.edu/irb/Doc/0/SL27V4LANKH490NTK8CTGGE433/MBSR%20Class%20Eight%20Measures.docx) \| \| [MBSR Class Four Questionnaires](https://sparcs.andrew.cmu.edu/irb/Doc/0/RJVIQE75F6J4D9K09AD89UR00A/MBSR%20Class%20Four%20Measures.docx) \| \| [MBSR Class One Questionnaires](https://sparcs.andrew.cmu.edu/irb/Doc/0/O10IP8CT6SPKJFOTSUDBDFFCD9/MBSR%20Class%20One%20Measures.docx) \| \| [Post Session Post-TSST Questionnaire](https://sparcs.andrew.cmu.edu/irb/Doc/0/E4BF37QQ8G84T11UC0SB4Q5F25/SMART%20Post-TSST%20Questionnaire%20Packet%2010-15-15.docx) \| \| [Post Session Post-TSST Thought Listing Questionnaire](https://sparcs.andrew.cmu.edu/irb/Doc/0/75UM777BMCEKV769CU36TFMRFA/Thought%20Listing.docx) \| \| [Post Session Pre-TSST Thought Listing Questionnaire](https://sparcs.andrew.cmu.edu/irb/Doc/0/CRQ0SJ62RIGKN18TH7D9U89G26/SMART%20Pre-TSST%20Thought%20Listing.docx) \| \| [Post Session Program Evaluation](https://sparcs.andrew.cmu.edu/irb/Doc/0/V1UD43I4ARO4N1UD38ELA65937/SMART%20Program%20Evaluation.docx) \| \| [Post Session Questionnaire](https://sparcs.andrew.cmu.edu/irb/Doc/0/2DEEGCV66MR4R4G80MSLHIICE1/SMART%20Post%20Questionnaire%20Packet.docx) \| \| \| --- \| --- \| --- \| --- \| --- \| --- \| --- \| --- \| --- \| --- \| --- \| --- \| \|  \| |
| \| \|  \| \| View: CMU-SF: External Sites \| \| --- \| \| \| --- \| --- \| --- \| \| \| --- \| --- \| --- \| --- \| |
| Research Locations and Collaborating Institutions   [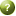](https://www.cmu.edu/research-office/sparcs/helptext/irb-help-text.pdf#nameddest=locationsandcollaborations)  **Research Locations**   1. *** a) Where will the research be conducted?  If the research will be conducted via the internet, select the location of the primary researcher.  Check all that apply:** **Pittsburgh campus** **Qatar campus** **Rwanda campus** **Silicon Valley campus** **Other**   * **b) If other, please indicate the location(s).**  **c) If you are conducting research on one of the CMU campuses indicated above, please specify the exact location.** Carnegie Mellon University 5000 Forbes Avenue Pittsburgh, PA 15213 Baker Hall Room 340J   1. **a) If conducting research on a non-CMU property, have you received permission to conduct research at this location?** [Yes](https://sparcs.andrew.cmu.edu/irb/ResourceAdministration/Project/PrintSmartForms?Project=com.webridge.entity.Entity%5BOID%5B8809FBF1C9DBD547B44CE3C93A19FFC2%5D%5D&rootEntity=com.webridge.entity.Entity%5BOID%5B8809FBF1C9DBD547B44CE3C93A19FFC2%5D%5D&PrintBySection=False&PrintHeaderView=False&PrintHeaderInfo=True&PrintPageBreak=True&PrintLogo=True&showHiddenData=False&showCDTFormsAtEnd=True)   **c) If applicable, please attach documentation of permission to conduct**  **research in your proposed location:**   \| \| Name \| \| --- \| \| [Phipps letter of support.docx](https://sparcs.andrew.cmu.edu/irb/Doc/0/TJ4B61HQGD1472KSC82BE8B441/Phipps%20letter%20of%20support.docx) \| \| \| --- \| --- \| --- \| \|  \|  1. *** a) In what country will the research be conducted (check all that apply)?** **United States** **Qatar** **Other**   **Collaborations**   1. * **a) Is this research intended to be done in cooperation with any institutions, individuals or organizations not affiliated with CMU?**   **Yes** No   **b) List the collaborators involved with the study by clicking on the Add button:**   \| \|  \| Collaborators (from list) \| Collaborators (Manual Entry) \| Is CMU Overseeing Collaborator \| Is Collaborator Overseeing CMU \| \| --- \| --- \| --- \| --- \| --- \| \| [View](https://sparcs.andrew.cmu.edu/irb/ResourceAdministration/Project/PrintSmartForms?Project=com.webridge.entity.Entity%5BOID%5B8809FBF1C9DBD547B44CE3C93A19FFC2%5D%5D&rootEntity=com.webridge.entity.Entity%5BOID%5B8809FBF1C9DBD547B44CE3C93A19FFC2%5D%5D&PrintBySection=False&PrintHeaderView=False&PrintHeaderInfo=True&PrintPageBreak=True&PrintLogo=True&showHiddenData=False&showCDTFormsAtEnd=True) \|  \| Carol Greco and Deanna Burkett (UPMC Center for Integrative Medicine) \| no \| no \| \| [View](https://sparcs.andrew.cmu.edu/irb/ResourceAdministration/Project/PrintSmartForms?Project=com.webridge.entity.Entity%5BOID%5B8809FBF1C9DBD547B44CE3C93A19FFC2%5D%5D&rootEntity=com.webridge.entity.Entity%5BOID%5B8809FBF1C9DBD547B44CE3C93A19FFC2%5D%5D&PrintBySection=False&PrintHeaderView=False&PrintHeaderInfo=True&PrintPageBreak=True&PrintLogo=True&showHiddenData=False&showCDTFormsAtEnd=True) \|  \| Joshua Smyth (Pennsylvania State University) \| no \| no \| \| \| --- \| --- \| --- \| --- \| --- \| --- \| --- \| --- \| --- \| --- \| --- \| --- \| --- \| --- \| --- \| --- \| \|  \|   **c) Does CMU have responsibility for oversight of the entire study?**    **Yes** No  **d) Describe the monitoring process for the entity(ies) who will have responsibility for oversight of the study:**  Dr. Creswell will be responsible for oversight of the entire study.  **e) Is there IRB approval from another IRB for this study?** [No](https://sparcs.andrew.cmu.edu/irb/ResourceAdministration/Project/PrintSmartForms?Project=com.webridge.entity.Entity%5BOID%5B8809FBF1C9DBD547B44CE3C93A19FFC2%5D%5D&rootEntity=com.webridge.entity.Entity%5BOID%5B8809FBF1C9DBD547B44CE3C93A19FFC2%5D%5D&PrintBySection=False&PrintHeaderView=False&PrintHeaderInfo=True&PrintPageBreak=True&PrintLogo=True&showHiddenData=False&showCDTFormsAtEnd=True) |
| \| \|  \| \| View: CMU-SF: Study Deception \| \| --- \| \| \| --- \| --- \| --- \| \| \| --- \| --- \| --- \| --- \| |
| Deception [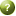](https://www.cmu.edu/research-office/sparcs/helptext/irb-help-text.pdf#nameddest=studydecep)  Deception is only possible in minimal risk studies. Investigators need to explain why the deception is necessary to achieve the study goals and how the degree of deception is kept to a minimum. The degree of deception means, for example, withholding part of the study’s purpose as opposed to stating a false study purpose. Subjects should be debriefed as early as is feasible.   1. * **a) Will deception be used?** Yes   **b) If yes, please explain:** Participants will not be aware of the exact nature of the attention training conditions or our study hypotheses, but they will be informed of our aims at the completion of the study. Participants will be fully debriefed on the nature of the tasks. **c) If yes, please include a justification as to why deception is necessary:** Deception is used to improve attention and task effort. **d) If yes, describe how participants will be debriefed:** Participants are fully debriefed by a research assistant who leads the entire final study session. Research assistants use the script attached to explain study hypotheses and reasons for deception, and are trained to answer additional questions and tend to participants' emotional reactions to ensure that the participant is not upset when they leave.  **e) Please include the de-briefing material and/or script:** [De-briefing Packet(0.01)](https://sparcs.andrew.cmu.edu/irb/Doc/0/T5S1V74CT26K932DELMA85DSC6/R21_debrief_10-15-15.docx) |
| \| \|  \| \| View: CMU-SF: Participant Info (All) \| \| --- \| \| \| --- \| --- \| --- \| \| \| --- \| --- \| --- \| --- \| |
| Participant Information [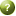](https://www.cmu.edu/research-office/sparcs/helptext/irb-help-text.pdf#nameddest=participantinfo)   1. * **What is the age range of participants in the proposed study?** 18+ 2. * **How many participants are needed for the study?** 160 3. * **How was that number determined?** Based on previous research in the area 4. * **Please list inclusion and exclusion criteria:** Inclusion Criteria: a) English-speaking adults between the ages of 18 and 70 b) owns an internet-enable smartphone  Exclusion Criteria: a) report a new significant chronic mental (e.g., recurrent depression, schizophrenia, personality disorder) or physical (e.g., cancer, HIV, diabetes) disease, including bleeding disorders, in the last three months b) hospitalization in the last 3 months c) illicit drug use d) use of oral contraceptives (females only) e) pregnant (females only) f) breastfeeding (females only) g) Existing mind-body stress reduction practice, specifically: 90 minutes or more each week of guided meditation, yoga, tai chi, journaling, or relaxation/meditation YouTube videos 5. * **a) What do you estimate the ratio of males to females to be?** 1:1  * **b) Will this be reflective of the local population?**   **Yes** No |
| \| \|  \| \| View: CMU-SF: Participant Info (Non-Exempt) \| \| --- \| \| \| --- \| --- \| --- \| \| \| --- \| --- \| --- \| --- \| |
| Participant Information (Non-Exempt) [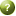](https://www.cmu.edu/research-office/sparcs/helptext/irb-help-text.pdf#nameddest=partinfononexempt)   1. * **Will vulnerable subjects (Pregnant Women, Neonates, Prisoners, Children, and Cognitively Impaired Adults) be involved in the proposed study?**   Yes **No** * **Pregnant women, human fetuses:** No  **Neonates:**   Yes **No Prisoners:**   Yes **No Children:**   Yes **No Cognitively impaired adults:**   Yes **No** 2. * **Will the participants be capable of understanding the nature of the study and the consent process?**   **Yes** No 3. * **Will you target a certain population?**   **Yes** No  * **Please explain:** Participants who indicate moderate to high levels of perceived stress in the past month during the initial telephone screening interview. 4. * **a) Do you anticipate that your participants will represent a cross-section of the population in the region where the study is being conducted?**   **Yes** No   **b) If yes, please describe and estimate the percentage that will be from minority groups:** Minority enrollment will roughly match the demographic distribution of the Pittsburgh community. |
| \| \|  \| \| View: CMU-SF: Recruitment \| \| --- \| \| \| --- \| --- \| --- \| \| \| --- \| --- \| --- \| --- \| |
| Recruitment [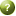](https://www.cmu.edu/research-office/sparcs/helptext/irb-help-text.pdf#nameddest=recruit)   1. * **Describe how participant recruitment will be performed:** To recruit community members, we will post newspaper/internet/bus/print ads/emails across the Pittsburgh community. We will also post the study on the University of Pittsburgh CTSI Research Participant Registry, Allegheny County Port Authority buses, the CMU Center for Behavioral Decision Research Participant Pool website, and the CMU Psychology Department Paid Experiment System. Participants can email or call our study hotline for more information and a pre-screening interview. 2. * **Indicate how and by whom potential participants are introduced to the study:** Trained research assistants and the study investigators will return calls and emails of interested potential participants. Second, we will be using the professional services of Scott Beacher at the University of Pittsburgh to recruit and phone screen potential participants. The University Center for Social & Urban Research maintains a database of 4,000 regional residents willing to participate in research. Potential participants screened eligible through UCSUR will be directed to the Health & Human Performance lab to complete the study. 3. **Check all boxes below that apply and attach documentation using Question 5:**  \| **Flyers?** \| **Where will they be posted?** \| \| --- \| --- \| \|  \| Carnegie Mellon campus, local organizations with ties to stressed populations \|  \| **Radio, TV?** \|  \| \| --- \| --- \| \|  \|  \|  \| **E-Mail?** \| **Indicate how the email addresses are obtained:** \| \| --- \| --- \| \|  \| If we can obtain consent from interested community list-serve administrators, we will ask them to send out a blast email to their listserves. \|  \| **Web-based?** \| **Specify Sites:** \| \| --- \| --- \| \|  \| CraigsList \|  \| **Participant Pool?** \| **Specify which pool is being used:** \| \| --- \| --- \| \|  \| University of Pittsburgh CTSI Research Participant Registry, the CMU Center for Behavioral Decision Research Participant Pool website, and the CMU Psychology Department Paid Experiment System, UCSUR Research Registry (http://ucsur.pitt.edu/ucsur-research-registry/) \|  \| **Other?** \| **Please specify:** \| \| --- \| --- \| \|  \| Local newspaper and bus ads \|  1. * **Will participants undergo screening prior to their participation?**   **Yes** No **If yes, please describe:** Yes, participants will undergo a 10-minute telephone screening during which we will explain the study in greater depth and administer a screening questionnaire consistent with the inclusion and exclusion criteria specified in the Participant Information section; additionally, all participants must report a significant amount of stress in the past month. 2. **Please attach all recruiting and screening materials:**  \| \|  \| Document \| Category \| Date Modified \| Document History \| \| --- \| --- \| --- \| --- \| --- \| \| [View](https://sparcs.andrew.cmu.edu/irb/ResourceAdministration/Project/PrintSmartForms?Project=com.webridge.entity.Entity%5BOID%5B8809FBF1C9DBD547B44CE3C93A19FFC2%5D%5D&rootEntity=com.webridge.entity.Entity%5BOID%5B8809FBF1C9DBD547B44CE3C93A19FFC2%5D%5D&PrintBySection=False&PrintHeaderView=False&PrintHeaderInfo=True&PrintPageBreak=True&PrintLogo=True&showHiddenData=False&showCDTFormsAtEnd=True) \| [Phone Screening Script(2)](https://sparcs.andrew.cmu.edu/irb/Doc/0/01NM2RNM7LD45F9KJSFN1EOG1D/SMART_Phone%20Screening_2-13-15.docx) \| Recruitment Materials \| 7/14/2016 \| [History](https://sparcs.andrew.cmu.edu/irb/ResourceAdministration/VersionedResource/MetaInfoViewer?bindTo=com.webridge.entity.Entity%5bOID%5b6DFE65B615CAAF44AA21FC5B9B9C5B3F%5d%5d&Document=com.webridge.entity.Entity%5bOID%5b6DFE65B615CAAF44AA21FC5B9B9C5B3F%5d%5d) \| \| [View](https://sparcs.andrew.cmu.edu/irb/ResourceAdministration/Project/PrintSmartForms?Project=com.webridge.entity.Entity%5BOID%5B8809FBF1C9DBD547B44CE3C93A19FFC2%5D%5D&rootEntity=com.webridge.entity.Entity%5BOID%5B8809FBF1C9DBD547B44CE3C93A19FFC2%5D%5D&PrintBySection=False&PrintHeaderView=False&PrintHeaderInfo=True&PrintPageBreak=True&PrintLogo=True&showHiddenData=False&showCDTFormsAtEnd=True) \| [SMART_recruitmentflyer_6-15-15.doc(0.01)](https://sparcs.andrew.cmu.edu/irb/Doc/0/TGVAKNQHPDQ431EDNT406BT823/SMART_recruitmentflyer_6-15-15.doc) \| Recruitment Materials \| 3/7/2016 \| [History](https://sparcs.andrew.cmu.edu/irb/ResourceAdministration/VersionedResource/MetaInfoViewer?bindTo=com.webridge.entity.Entity%5bOID%5b89EC71264B49ED48832FCFE0AB87D0EC%5d%5d&Document=com.webridge.entity.Entity%5bOID%5b89EC71264B49ED48832FCFE0AB87D0EC%5d%5d) \| \| [View](https://sparcs.andrew.cmu.edu/irb/ResourceAdministration/Project/PrintSmartForms?Project=com.webridge.entity.Entity%5BOID%5B8809FBF1C9DBD547B44CE3C93A19FFC2%5D%5D&rootEntity=com.webridge.entity.Entity%5BOID%5B8809FBF1C9DBD547B44CE3C93A19FFC2%5D%5D&PrintBySection=False&PrintHeaderView=False&PrintHeaderInfo=True&PrintPageBreak=True&PrintLogo=True&showHiddenData=False&showCDTFormsAtEnd=True) \| [Recruitment Flyer(0.01)](https://sparcs.andrew.cmu.edu/irb/Doc/0/H88LI80BRF9KRALD7L2QK5AQ02/SMART_recruitmentflyer_6-15-15.pdf) \| Recruitment Materials \| 3/7/2016 \| [History](https://sparcs.andrew.cmu.edu/irb/ResourceAdministration/VersionedResource/MetaInfoViewer?bindTo=com.webridge.entity.Entity%5bOID%5b28E267632D9B754FA2DBBCDBC73694CB%5d%5d&Document=com.webridge.entity.Entity%5bOID%5b28E267632D9B754FA2DBBCDBC73694CB%5d%5d) \| \| \| --- \| --- \| --- \| --- \| --- \| --- \| --- \| --- \| --- \| --- \| --- \| --- \| --- \| --- \| --- \| --- \| --- \| --- \| --- \| --- \| --- \| \|  \| |
| \| \|  \| \| View: CMU-SF: Consent Info (Non-Exempt) \| \| --- \| \| \| --- \| --- \| --- \| \| \| --- \| --- \| --- \| --- \| |
| Consent (Non-Exempt) [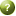](https://www.cmu.edu/research-office/sparcs/helptext/irb-help-text.pdf#nameddest=consentnonexempt)   1. * **a) Do you plan to use consent forms?**   **Yes** No   [**Link to CMU Consent Form Templates**](https://www.cmu.edu/research-compliance/human-subjects-research/guidance-forms.html)  **b) If yes, describe the process of how consent will be obtained, and by whom:** At the start of the initial baseline visit at our laboratory at Carnegie Mellon University, a trained research assistant will administer and review the paper consent form with the potential participant. All potential participants are given the time they need to review the consent form to decide whether or not they would like to participate in the study.  **c) If yes, please attach your consent form(s) here:**   \| \|  \| Document \| Category \| Date Modified \| Document History \| \| --- \| --- \| --- \| --- \| --- \| \| [View](https://sparcs.andrew.cmu.edu/irb/ResourceAdministration/Project/PrintSmartForms?Project=com.webridge.entity.Entity%5BOID%5B8809FBF1C9DBD547B44CE3C93A19FFC2%5D%5D&rootEntity=com.webridge.entity.Entity%5BOID%5B8809FBF1C9DBD547B44CE3C93A19FFC2%5D%5D&PrintBySection=False&PrintHeaderView=False&PrintHeaderInfo=True&PrintPageBreak=True&PrintLogo=True&showHiddenData=False&showCDTFormsAtEnd=True) \| [SMART Study Consent Form(2)](https://sparcs.andrew.cmu.edu/irb/Doc/0/30GQDO5CR7P4FAHU0DFS4NMA13/R21_consent_form_9-21-15.doc) \| Consent Form \| 7/14/2016 \| [History](https://sparcs.andrew.cmu.edu/irb/ResourceAdministration/VersionedResource/MetaInfoViewer?bindTo=com.webridge.entity.Entity%5bOID%5b00E470F7F9F9D44D8BEAEDAD586CC676%5d%5d&Document=com.webridge.entity.Entity%5bOID%5b00E470F7F9F9D44D8BEAEDAD586CC676%5d%5d) \| \| \| --- \| --- \| --- \| --- \| --- \| --- \| --- \| --- \| --- \| --- \| --- \| \|  \|  1. **Will the consent form be presented on paper?**   **Yes** No 2. **Will the consent form be presented online?**   Yes **No** 3. **Are you requesting to use a consent form that is different from the CMU model consent?**   Yes **No**   **Waiver of Informed Consent**  If requesting a waiver of informed consent, please complete the following:   1. * **a) Are you requesting a waiver of informed consent?**   Yes **No**   **Waiver of Written Consent  If requesting a waiver of written consent, please complete the following:**   1. * **a) Are you requesting a waiver of written (signed) documentation of informed consent?**   **Yes** No   **b) If yes, please indicate which one of the following applies:** The only record linking the subject and the research would be the consent document and the principal risk would be potential harm resulting from a breach of confidentiality.  **c) Explain how the study meets the criteria above:** We request a waiver of written (signed) informed consent for the initial phone screening. In this phone screening we verbally describe all aspects of the study and request prospective participants' verbal consent to provide answers to our screening questions. Specifically, prior to screening, the participant will be told "You may feel uncomfortable answering some of the questions about your medical history and personal life. You do not have to answer any questions you do not wish to answer and you may stop at any time. Your participation in the screening is voluntary. A decision whether or not to participate in the screening will not affect your relationship with Carnegie Mellon University. You will not directly benefit from the screening. Your answers will be confidential. We intend to use the screening information to determine if you qualify for the study. If you do not qualify for the study, your answers will be destroyed. If you do qualify for the study, elect to participate, and provide informed consent, we will keep your answers as part of your research data for the duration of the study in a locked cabinet." Our verbal consent to screen involves no more than minimal risk to subjects. The screening script is included in our submission materials.  **d) Is the waiver for all study participants?**   **Yes** No  **f) Is the waiver for all study procedures?**   Yes **No**  **g) If no, to what procedures does the waiver apply?** The waiver is only for the initial phone screening.  **Minor Participation  If participants are minors, please complete the following:**   1. **Please describe how assent will be obtained:** 2. **Are minors at a developmentally appropriate age to assent?  Please describe how this was determined:** |
| \| \|  \| \| View: CMU-SF: Risk and Benefits (All) \| \| --- \| \| \| --- \| --- \| --- \| \| \| --- \| --- \| --- \| --- \| |
| Risk and Benefits (All)  [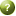](https://www.cmu.edu/research-office/sparcs/helptext/irb-help-text.pdf#nameddest=risksall)   1. * **Will participants receive a direct benefit from the study?**   **Yes** No  **Discuss the expected direct benefits to participants:** Participants may benefit by learning about psychological research. They may also find the attention trainings to be useful and decide to continue practicing upon completion of the study. 2. * **Discuss the expected indirect benefits to participants:** Participants may benefit by learning about psychological research. They may also find the attention trainings to be useful and decide to continue practicing upon completion of the study. 3. * **Discuss the potential risks to participants:** Potential risks include breach of confidentiality since we will be obtaining participants' email addresses and signatures. This risk will be minimized in several ways. Participants' email addresses will be stored in our password-protected laboratory email account and linked only to participant information by study ID number. A file linking study ID numbers to personal identifying information will be kept in a locked file cabinet in a locked room to which only a member of the Health and Human Performance Lab at Carnegie Mellon University will have access. Participant signatures will be stored in a separate locked file cabinet in the locked Health and Human Performance Lab as well, and will not be linked with other personal identifying information.  It is possible that the blood spot collection procedure will be associated with slight pain from the lancet prick, though most people find the procedure painless. In rare cases (less than 1 percent), fainting at the sight of blood or infection may occur. All procedures will be done using sterile procedures by trained research staff to minimize these risks.   Potential risks also include possible discomfort or inconvenience from completing the questionnaires. If participants are uncomfortable answering any of the questions on the questionnaires or while completing any of the tasks, they may decline to answer those questions and/or discontinue their participation in the tasks.   Participants will also complete a laboratory challenge task in which they perform difficult tasks in front of an evaluative audience. This may cause anxiety in participants. The purpose of this task will be clearly explained to the participant during the debriefing, and all of their questions and concerns will be addressed. It will be made clear that the subject did not actually perform poorly on the task, and that the people giving them negative feedback was a part of our study. All efforts will be made to ensure the participant is not upset when they leave. We have used this experimental paradigm and debriefing method in many IRB-approved studies in our laboratory.  Some participants may find the performance tasks distressing when performing them. The participant will be informed that, if this does occur during any portion of the performance section of the study, they may decline to participate in this portion of the study or in the entire study all together. 4. * **Discuss how all potential risks will be managed and/or minimized:** See above. |
| \| \|  \| \| View: CMU-SF: Risk and Benefits (Non-Exempt) \| \| --- \| \| \| --- \| --- \| --- \| \| \| --- \| --- \| --- \| --- \| |
| Risk and Benefits (Non-Exempt)  [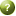](https://www.cmu.edu/research-office/sparcs/helptext/irb-help-text.pdf#nameddest=risksnonexempt)   1. **Is deception used?** Yes   Note: you answered this question on the Study Deception form.  If you need to change your response, use the jump menu above to go to the Study Deception smart form.   1. * **Indicate the degree of any possible (e.g., physical or psychological) risk you believe the research will pose to human subjects*:** Minimal Risk 2. * **Describe how the study fits in the selected risk level.   If deception is used in the study (see question 1 above), please address how it fits in the selected risk level:** Study activities are similar to experiences in everyday adult life.   *Risk Descriptions:  Minimal Risk: A risk is minimal where the probability and magnitude of harm or discomfort anticipated in the proposed research are not greater, in and of themselves, than those ordinarily encountered in daily life of during the performance of routine physical or psychological examinations or tests.   Greater than Minimal Risk: A risk is greater than minimal where the probability and magnitude of harm or discomfort anticipated in the proposed research are greater than those ordinarily encountered in daily life or during the performance of routine physical or psychological examinations or tests. |
| \| \|  \| \| View: CMU-SF: Compensation (All) \| \| --- \| \| \| --- \| --- \| --- \| \| \| --- \| --- \| --- \| --- \| |
| Compensation [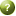](https://www.cmu.edu/research-office/sparcs/helptext/irb-help-text.pdf#nameddest=compall)   1. * **a) Are participants to be compensated for the study?**   **Yes** No   **b) If yes, what is the amount of compensation:** pro-rated $8-12/hr.$20 - first lab visit.$15 for <16 pre EMA surveys, $20 for 16, $25 for 17, & $30 for 18. $50-$125 depends on # of classes. $45 for <16 post EMA surveys, $60 for 16, $65 for 17, & $70 for 18. $50 - final lab visit. Extra travel.  **c) If yes, what is the source of the compensation:** NCCIH  **d) If yes, what is the type of compensation (eg, gift card, cash):** cash, gift card   1. * **Will participants receive any non-monetary compensation?**   Yes **No** 2. * **Are there any costs to participants?**   **Yes** No **If yes, please describe:** Transportation to CMU's campus for the first and last study visit; smartphone data plan that participants already pay for regardless of their participation in the study. |
| \| \|  \| \| View: CMU-SF: Compensation (Non-Exempt) \| \| --- \| \| \| --- \| --- \| --- \| \| \| --- \| --- \| --- \| --- \| |
| Compensation (Non-Exempt) [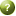](https://www.cmu.edu/research-office/sparcs/helptext/irb-help-text.pdf#nameddest=compnonexempt)   1. * **Will you compensate participants for injury resulting from participation?** [No](https://sparcs.andrew.cmu.edu/irb/ResourceAdministration/Project/PrintSmartForms?Project=com.webridge.entity.Entity%5BOID%5B8809FBF1C9DBD547B44CE3C93A19FFC2%5D%5D&rootEntity=com.webridge.entity.Entity%5BOID%5B8809FBF1C9DBD547B44CE3C93A19FFC2%5D%5D&PrintBySection=False&PrintHeaderView=False&PrintHeaderInfo=True&PrintPageBreak=True&PrintLogo=True&showHiddenData=False&showCDTFormsAtEnd=True) 2. **a) Will participants who are students be offered class credit?** No |
| \| \|  \| \| View: CMU-SF: Data Security and Confidentiality \| \| --- \| \| \| --- \| --- \| --- \| \| \| --- \| --- \| --- \| --- \| |
| Data Security and Confidentiality   [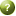](https://www.cmu.edu/research-office/sparcs/helptext/irb-help-text.pdf#nameddest=datasecurity)   1. * **Will personal identifiers be obtained?**   **Yes** No **If yes, list the personal identifiers:** Name, email address, telephone number, demographics 2. * **Describe your procedure for coding your data (encoding):** Each subject will be assigned a study ID number. A file linking study ID numbers to personal identifying information will be kept in a locked file cabinet in a locked room to which only a member of the Health and Human Performance Lab at Carnegie Mellon University will have access. All coded participant records will be stored in a separate locked cabinet in the Health and Human Performance Lab. Survey information will be identified by participants' study ID only. Saliva samples and physiological data will only be labeled with participant's unique study ID numbers. 3. * **Will audio recordings be made?**   Yes **No** 4. * **Will video recordings be made?**   **Yes** No **If yes, please describe:** We will record participants during the 10-minute laboratory performance task for later coding of facial affect. 5. * **Do you intend to obtain a certificate of confidentiality from NIH?**   Yes **No** 6. * **In addition to the individuals listed on the study personnel page, who will have access to research data (e.g. surveys, questionnaires, recordings, interview records, etc.)?** The PIs and other research staff will have access to the data. If participants give consent, their videos (but no other identifying information) will be uploaded to Mechanical Turk, an online workforce, where multiple workers will code aspects of the videos. 7. * **Describe how you will protect participant confidentiality and secure research records (e.g. password protected, encrypted, etc.). Include location of where the data will be stored:** A file linking study ID numbers to personal identifying information will be kept in a locked file cabinet in a locked room to which only a member of the Health and Human Performance Lab at Carnegie Mellon University will have access. All coded participant records will be stored in a separate locked cabinet in the Health and Human Performance Lab. Survey information collected on password protected laboratory computers will remain in our locked laboratory room, and survey information collected from smartphones will be uploaded automatically to our secure study website database; this information will be identified by participants' study ID only. Saliva samples and physiological data will only be labeled with participant's unique study ID numbers. Once collected, the saliva samples will be stored in a freezer within a locked room of the Health and Human Performance Lab; upon completion of the study saliva samples will be shipped for analyses of cortisol levels and then destroyed. 8. * **Describe your process for overseeing your study.   Include a description regarding monitoring of data (to ensure that study goals are met and adherence to the IRB approved protocol is maintained). Examples: Review of lab notebooks, frequency of meetings to review data, who will be present at the meetings, how recruitment and retention will be monitored, etc.:** In the beginning of the study a list of participant ID numbers and their specific conditions will be created in order to keep track of how many individuals are participating. Every week, one of the investigators will examine the number of participants who provided usable data for this experiment in order to determine how many participants are still needed. Staff in the lab will be trained to enter the data into SPSS & data entry will proceed as the data is collected. 9. * **Describe your process for ensuring that adverse events, unanticipated problems, and subject complaints are reported to the IRB Office in a timely manner:** All research assistants are trained with a protocol for managing adverse events. Participant safety is top priority; the phone number for campus police is posted in all lab rooms for use in the event of an emergency. The PIs will be contacted immediately after (or preferably during, if possible) any unanticipated event occurs. Research assistants will work with PIs to ensure that participants are not upset when they leave Baker Hall. If an adverse event does occur, PIs will meet promptly and report to the IRB within 24 hours. 10. **Confirm that all research data will be retained at CMU for a minimum of three (3) years past study completion:**  |
| \| \|  \| \| View: CMU-SF: Supporting Documents \| \| --- \| \| \| --- \| --- \| --- \| \| \| --- \| --- \| --- \| --- \| |
| Supporting Documents [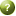](https://www.cmu.edu/research-office/sparcs/helptext/irb-help-text.pdf#nameddest=supportdocs)   1. **Attach supporting files:**  \| \|  \| Document \| Category \| Date Modified \| Document History \| \| --- \| --- \| --- \| --- \| --- \| \| [View](https://sparcs.andrew.cmu.edu/irb/ResourceAdministration/Project/PrintSmartForms?Project=com.webridge.entity.Entity%5BOID%5B8809FBF1C9DBD547B44CE3C93A19FFC2%5D%5D&rootEntity=com.webridge.entity.Entity%5BOID%5B8809FBF1C9DBD547B44CE3C93A19FFC2%5D%5D&PrintBySection=False&PrintHeaderView=False&PrintHeaderInfo=True&PrintPageBreak=True&PrintLogo=True&showHiddenData=False&showCDTFormsAtEnd=True) \| [Recruitment Flyer(0.01)](https://sparcs.andrew.cmu.edu/irb/Doc/0/GR7HVSO12234F09QQ29JE26ICC/SMART_recruitmentflyer_6-15-15.pdf) \| Other \| 2/19/2016 \| [History](https://sparcs.andrew.cmu.edu/irb/ResourceAdministration/VersionedResource/MetaInfoViewer?bindTo=com.webridge.entity.Entity%5bOID%5bB9CBBCF9926152478C381D997363EDF0%5d%5d&Document=com.webridge.entity.Entity%5bOID%5bB9CBBCF9926152478C381D997363EDF0%5d%5d) \| \| [View](https://sparcs.andrew.cmu.edu/irb/ResourceAdministration/Project/PrintSmartForms?Project=com.webridge.entity.Entity%5BOID%5B8809FBF1C9DBD547B44CE3C93A19FFC2%5D%5D&rootEntity=com.webridge.entity.Entity%5BOID%5B8809FBF1C9DBD547B44CE3C93A19FFC2%5D%5D&PrintBySection=False&PrintHeaderView=False&PrintHeaderInfo=True&PrintPageBreak=True&PrintLogo=True&showHiddenData=False&showCDTFormsAtEnd=True) \| [Questionnaire Sample(0.01)](https://sparcs.andrew.cmu.edu/irb/Doc/0/LJOKN4UH6EK41DB6GQQ6F0I55C/R21_questionnaire_sample_2-13-15%5B1%5D.doc) \| Questionnaire/Survey \| 2/19/2016 \| [History](https://sparcs.andrew.cmu.edu/irb/ResourceAdministration/VersionedResource/MetaInfoViewer?bindTo=com.webridge.entity.Entity%5bOID%5bBA5E4D828467EE44BD9021F94EB4C1D1%5d%5d&Document=com.webridge.entity.Entity%5bOID%5bBA5E4D828467EE44BD9021F94EB4C1D1%5d%5d) \| \| [View](https://sparcs.andrew.cmu.edu/irb/ResourceAdministration/Project/PrintSmartForms?Project=com.webridge.entity.Entity%5BOID%5B8809FBF1C9DBD547B44CE3C93A19FFC2%5D%5D&rootEntity=com.webridge.entity.Entity%5BOID%5B8809FBF1C9DBD547B44CE3C93A19FFC2%5D%5D&PrintBySection=False&PrintHeaderView=False&PrintHeaderInfo=True&PrintPageBreak=True&PrintLogo=True&showHiddenData=False&showCDTFormsAtEnd=True) \| [Phipps Conservatory Letter of Support(0.01)](https://sparcs.andrew.cmu.edu/irb/Doc/0/8DDL8FNV4UI4PF3H3BI37BIQ92/Phipps%20letter%20of%20support.docx) \| Site Permissions \| 2/19/2016 \| [History](https://sparcs.andrew.cmu.edu/irb/ResourceAdministration/VersionedResource/MetaInfoViewer?bindTo=com.webridge.entity.Entity%5bOID%5b28207283F8C45643A0192E912A3A5E75%5d%5d&Document=com.webridge.entity.Entity%5bOID%5b28207283F8C45643A0192E912A3A5E75%5d%5d) \| \| \| --- \| --- \| --- \| --- \| --- \| --- \| --- \| --- \| --- \| --- \| --- \| --- \| --- \| --- \| --- \| --- \| --- \| --- \| --- \| --- \| --- \| \|  \| |
